# Molecular Pathways and Pigments Underlying the Colors of the Pearl Oyster *Pinctada margaritifera* var. *cumingii* (Linnaeus 1758)

**DOI:** 10.3390/genes12030421

**Published:** 2021-03-15

**Authors:** Pierre-Louis Stenger, Chin-Long Ky, Céline Reisser, Julien Duboisset, Hamadou Dicko, Patrick Durand, Laure Quintric, Serge Planes, Jeremie Vidal-Dupiol

**Affiliations:** 1 IFREMER, UMR 241 Écosystèmes Insulaires Océaniens, Labex Corail, Centre Ifremer du Pacifique, BP 49, 98725 Tahiti, France; Pierrelouis.stenger@gmail.com (P.-L.S.); chinky@ifremer.fr (C.-L.K.); Celine.Reisser@ifremer.fr (C.R.); 2 IHPE, Univ Montpellier, CNRS, IFREMER, Univ Perpignan Via Domitia, Montpellier, France; 3 MARBEC, Univ Montpellier, CNRS, IFREMER, IRD, Montpellier, France; 4 Aix Marseille Univ, CNRS, Centrale Marseille, Institut Fresnel, Marseille, France; julien.duboisset@fresnel.fr (J.D.); hamadou.dicko@fresnel.fr (H.D.); 5 Service de Bioinformatique, Département IRSI, IFREMER, ZI de la Pointe du Diable, 29280 Plouzané, France; Patrick.Guido.Durand@ifremer.fr (P.D.); Laure.Quintric@ifremer.fr (L.Q.); 6 EPHE-UPVD-CNRS, USR 3278 CRIOBE, Labex Corail, PSL Research University, Université de Perpignan, Perpignan, France; planes@univ-perp.fr

**Keywords:** *Pinctada margaritifera*, color, pigment, transcriptomics, Raman spectroscopy

## Abstract

The shell color of the Mollusca has attracted naturalists and collectors for hundreds of years, while the molecular pathways regulating pigment production and the pigments themselves remain poorly described. In this study, our aim was to identify the main pigments and their molecular pathways in the pearl oyster *Pinctada margaritifera*—the species displaying the broadest range of colors. Three inner shell colors were investigated—red, yellow, and green. To maximize phenotypic homogeneity, a controlled population approach combined with common garden conditioning was used. Comparative analysis of transcriptomes (RNA-seq) of *P. margaritifera* with different shell colors revealed the central role of the heme pathway, which is involved in the production of red (uroporphyrin and derivates), yellow (bilirubin), and green (biliverdin and cobalamin forms) pigments. In addition, the Raper–Mason, and purine metabolism pathways were shown to produce yellow pigments (pheomelanin and xanthine) and the black pigment eumelanin. The presence of these pigments in pigmented shell was validated by Raman spectroscopy. This method also highlighted that all the identified pathways and pigments are expressed ubiquitously and that the dominant color of the shell is due to the preferential expression of one pathway compared with another. These pathways could likely be extrapolated to many other organisms presenting broad chromatic variation.

## 1. Introduction

Color is a well-known trait involved in many biological interactions in nature, but the eye-catching color range of some gem-producing Mollusca such as pearl oysters and abalones has also attracted human interest. Mollusca is the largest marine phylum when considering the number of species [1]. Most of these animals have a shell displaying incredible colors and patterns of pigmentation. However, much remains to be discovered about the nature of these pigments and the molecular pathways that produce these colors [2]. Such knowledge could have widespread implications from evolutionary biology to economics [2,3].

Shell color can have a broad range of origins, ranging from a pure genetic basis to a pure environmental one [4,5,6]. Abalones are a textbook illustration of such a panel of drivers. In *Haliotis discus hannai* (Ino, 1953), the bluish and greenish colors are determined genetically by the combination between a recessive and a dominant allele [4]. However, in *Haliotis rufescens* (Swainson, 1822), diet determines the color expressed [6,7]—the reddish coloration is due to the uptake of red algae [8]. The phycoerythrin pigment contained in red algae is metabolized (tetrapyrrole synthesis in plants pathway) by the abalone into the red bile pigment rufescine [9]. Carotenoid pigments (astaxanthin, β-carotene, and cantaxanthin) are also known to contribute to this coloration, although the drivers controlling the expression of these additional pigments are unknown [6]. The mechanisms behind the characteristic reddish color of *H. rufescens* are therefore convoluted, highlighting the complexity of identifying both the pigments involved and the pathways by which they are produced.

To address this challenge, analytical chemistry [10,11,12] and transcriptomic analyses [13,14,15,16] can offer complementary approaches to identify pigments and pigment-producing pathways. Lemer et al., 2015 [17] performed a comparative transcriptomic analysis of black and albino pearl oysters *P. margaritifera* (Linnaeus, 1758) and identified putative pigmentation-related genes (*shem 4*, *mp8*, *krmp*, *chit*, and *serp*) involved in the synthesis of black eumelanin, the dominant color in this species. Analytical chemistry techniques were used to study the pigments responsible for its broad range of color. Chromatography and spectroscopic analyses revealed the important role of porphyrins (a group of heterocyclic macrocycle organic compounds) in the pigmentation of several species of the *Pinctada* genus. Uroporphyrin, which can lead to red or purple coloration [10,12], was present in black cultured pearls, as well as in the nacre (inner shell) and the prismatic layer (outer shell) of *P. margaritifera* [18]. Its presence was also confirmed more recently using Raman spectroscopy [19]. The use of Ultraviolet Visible (UV-Vis) spectrophotometry and physicochemical approaches has indicated that red, yellow, brown, and black coloration of *P. margaritifera* may result from an “unusual” melanin [20], or from a combination of eumelanin and pheomelanin [21]. While these results are essential starting points in understanding shell coloration, the joint identification of pigments and molecular pathways involved in the production of color is now necessary in order to improve our understanding of these complex mechanisms and provide innovative tools for commercial pearl production.

**Figure 1:**
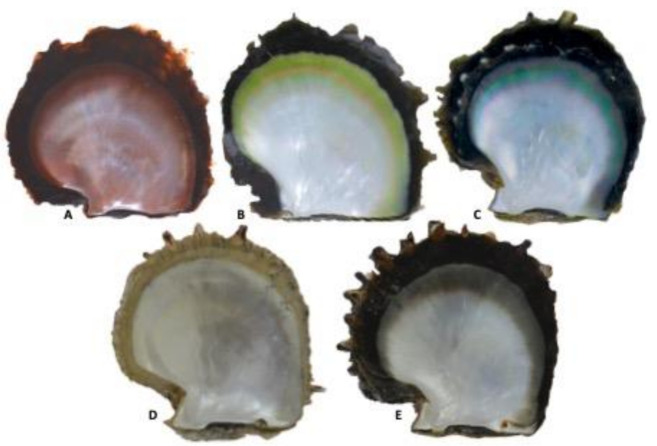
Different colors of *P. margaritifera* inner shell: (**A**): red, (**B**): yellow, (**C**): green, (**D**): albino, (**E**): black.

In this study, our aim was to identify the main pigments and pigment-producing pathways likely responsible for three economically major inner shell colors in *P. margaritifera*: red [22], yellow [22], and green [23] (Figure 1). Color-specific populations were farmed and reared in a common garden to limit confounding environmental effects. Their transcriptomes were compared by an RNA-seq approach to reveal differential gene expression among different pigment-producing pathways. The presence of the hypothetic pigments we identified from this transcriptomic approach was tested using Raman spectroscopy on the shells, which permits a direct and specific chemical characterization method. The joint use of these two methods led us to confidently identify both the pigments and the pathways underlying pearl oyster inner shell color phenotypes.

## 2. Materials and Methods

### 2.1. Biological Material

To obtain homogeneous phenotypes with red, green, and yellow shells, three multi-parental reproduction (10 males and 10 females each) were made at the Regahiga Pearl farm (23°06′56.6″ S 134°59′08.4″ W, Mangareva island, Gambier archipelago, French Polynesia) following a previously described procedure [24,25]. After two years of growth (corresponding to the stage of maximum pigmentation expression [22]), 200 individuals of each population (with a dorsal to ventral shell measurement between 10 and 12 cm) were selected for their color and transferred to Ifremer’s experimental concession (Tahiti island, Society archipelago, French Polynesia: concession No. 8120/MLD: 17°48′39.0″ S 149°18′03.8″ W) following regulations of the Ministry of Marine Resources of French Polynesia (transfer authorization No. 3605). To reduce transcriptomic variability linked to environmental influences, all 600 individuals were maintained in a common garden (i.e., in the same area) for two months (October–November 2016, optimal growth season [26,27]). Finally, four individuals of each population displaying the strongest inner shell color were selected and immediately dissected. A piece of mantle corresponding to the part use for grafting was sampled as previously described [28] and stored in RNAlater™ (4 °C for 24 h then −80 °C).

### 2.2. RNA Extraction, Purification, and Sequencing

Mantle tissue samples were individually ground in liquid nitrogen in a Retsh^®^ MM400 grinder (grinding speed = 30 oscillations/sec for 20 s) (Retsh, Haan, Germany). RNA extraction was performed using TRIZOL^®^ Reagent (Life Technologies™, Carlsbad, CA, USA) according to the manufacturer’s recommendations. After RNA precipitation, the pellets were suspended in RNA secure reagent^®^ (ThermoFisher Scientific, Waltham, MA, USA) and heated to 65 °C for 10 min to inactivate the RNase. DNA contamination was removed with the DNA-free kit (Ambion^®^ RNA Life Technologies™, Carlsbad, CA, USA) according to the manufacturer’s instructions. Finally, RNAs were cleaned with the PureLink™ RNA Mini Kit (Ambion^®^ RNA Life Technologies™™, Carlsbad, CA, USA) according to the manufacturer’s protocol. RNA quality and quantity were verified with a NanoDrop 1000© and an Agilent 2100 Bioanalyzer^®^ (Agilent Technologies™, Santa Clara, CA, USA). RNA sequencing libraries were produced using the Truseq3 kit. Sequencing was performed on an Illumina^®^ HiSeq^®^ 4000 (Illumina, San Diego, CA, USA), with 100 bp stranded paired-end reads. Library construction and sequencing were done by Génome Québec (Montreal, Québec, QC, Canada) (MPS Canada).

### 2.3. Bioinformatics Analysis

Analyses were performed at the ABIMS Roscoff Galaxy facility (galaxy3.sb-roscoff.fr; accessed on 22 January 2019). Raw data are available through the NCBI Sequence Read Archive (SRA, BioProject PRJNA521849, BioSample SUB5166470). Read quality was assessed using the FastQC program (V0.11.5) (www.bioinformatics.babraham.ac.uk/projects/fastqc; accessed on 22 January 2019). Raw reads were filtered with Trimmomatic V0.36.4 [29] to remove Illumina adapters (for Truseq3) and reads with an average Q-value below 26 for 95% of their length. To characterize and quantify the transcriptome of each sample, the filtered reads were paired-mapped against a *P. margaritifera* draft genome [30] with TopHat (V1.4.0). Cufflinks (V2.2.1.0) and Cuffmerge (V2.2.1.0) were used to assemble and merge the transcriptome produced for each library, respectively [31]. HTSeq-count (V0.6.1) [32] was used to obtain read count per transcript. All codes and parameters used for bioinformatics analysis are given in Supplementary Materials Table S1.

### 2.4. Transcriptome Functional Annotation

The transcriptome produced was annotated by sequence comparison against worldwide databases. First, an initial annotation with PLASTX [33] was made against NR data base (*e*-value at 1 × 10^−3^) [34] and Uniprot-Swissprot (*e*-value at 1 × 10^−3^) [35]. A protein domain search was then performed with InterProscan [36]. Finally, Gene Ontology terms were assigned with Blast2GO [37]. Scripts are provided in Supplementary Materials Table S1.

### 2.5. Differential Molecular Function and Gene Expression

To analyze our data, we followed a two-step strategy. The first step was transcriptome-wide, considering the entire transcriptome for each color, and using RBGOA tool [38] to identify significantly over-, or under-represented molecular function. To weigh the analysis, the -Log(*p*-value) method was used to take into account the strength and significance of the regulation of each gene of the transcriptome (https://github.com/z0on/GO_MWU; accessed on 22 January 2019). The second step of the strategy was more targeted and aimed to identify candidate genes directly from significantly Differentially Expressed Genes (DEGs). For each approach, we performed the same pairwise comparisons (red vs. yellow, red vs. green, and yellow vs. green) using the DESeq2 R package (v. 3.7) [39]. The three color phenotypes were used at the same factor level (green individuals compared against yellow individuals compared against red individuals). The collective gene expression differences between phenotypes were examined with a Principal Component Analysis (PCA) from the tool set of the *DESeq2* R package (v. 3.7) [39]. Gene expression differences between color phenotypes obtained with DEseq2, were considered significant below the 5% level (adjusted *p*-value (Padj) for multiple testing with the Benjamini-Hochberg procedure FDR *<* 0.05). The online version of KAAS (http://www.genome.jp/tools/kaas/; accessed on 23 June 2018) was used to find the functional pathways in which significant DEGs were involved [40]. Pathview (https://bioconductor.org/packages/release/bioc/html/pathview.html; accessed on 27 July 2018) was used to link differential expression with the KEGG Automatic Annotation Server (KAAS) pathways.

### 2.6. Enzymatic Structure Analysis by Homology Modeling

Three-dimensional homology modeling of the protein structures (longest ORF) was performed on the PDB (Protein Data Bank) file obtained with I-TASSER (https://zhanglab.ccmb.med.umich.edu/I-TASSER/; accessed on 30 March 2019) for the four PBGD sequences found in the DEGs analysis (named PBGD_1 to PBGD_D). The secondary structures are results from the I-TASSER analysis. Modeling was done with UCSF Chimera software [41]. Hinge residues were determined with the HingProt server link in Song et al. 2009 [42]. Superimposition of our candidate proteins on a reference protein with a known structure was performed in SuperPose [43] (Version 1, http://wishart.biology.ualberta.ca/SuperPose/; accessed on 2 July 2019). Only the longest PBGD (A) is shown. Detailed parameters are given in Supplementary Materials Table S2.

### 2.7. Raman Spectroscopy

Three individuals from the red, the yellow, and the green population were studied. Additionally, three black and three albino individuals were also studied and used as control. Raman spectra were acquired using a Raman spectrometer in reflection mode (LabRAM Evolution spectrometer, Horiba, Kyoto, Tokyo) with a 10× air objective (NA 0.4, Carl Zeiss, Oberkochen, Germany). The laser (632.8 nm) was focused on the colored border of the inner shell. The power delivered at the sample level was 1 mW on average. Three spectral windows, from 300 cm^−1^ to 1800 cm^−1^ (380–880 mm; 880–1380 mm; 1380–1880 mm), were recorded using an array of 1200 lines/mm. The acquisition time for each window was three hours.

### 2.8. Raman Spectra Analysis

Vibrational spectra of pearl oyster shell molecules were produced and analyzed by principal component analysis following the techniques described in Bonnier and Byrne, 2012 [44] directly on raw spectra. Then, subtraction of the baseline and a lightweight smoothing (5 points) were added to the raw spectra data. To improve data accuracy, spectra from each of the three windows were mathematically calibrated against referential inorganic components found in molluscan shell. Thus, calcite at 703 cm^−1^ was used for the 380–880 mm window [19], aragonite at 1085 cm^−1^ was used for the 880–1380 window [19], and carbonate at 1547 cm^−1^ was used for the 1380–1880 window [45]. The stat_peaks function from the *ggspectra* [46] R package (V. 0.3.5) was used to identify the exact peak position from the raw data to perform these calibrations. A manual verification was then made.

In order to associate peaks with pigments, all peaks from all spectra were extracted with the stat_peaks function from the *ggspectra* [46] R package (V. 0.3.5) and compared with a homemade bibliographic database (897 peaks obtained from 36 marine molluscan pigments extracted from 65 species in 32 studies, see Supplementary Materials Table S3). Since biological materials are known to produce noisy spectra, three different methods were used to compare peaks between the different shells analyzed [19,44,47]. First, a visual screening was operated to identify peaks matching or not expected signals. They were classified as: (*i*) *clear peak*, (*ii*) *putative peak*, and (*iii*) *no* peak. Second, the intensity of each selected peak was measured (the value of the lowest point of the peak minus the value of the peak). Third, a ratio was calculated between the intensity of the selected peak (calculated as previously described) and the peak of the referential calcite for each spectrum.

## 3. Results

### 3.1. Sequencing Results

Based on Illumina sequencing and after cleaning, 61,663,289 (±2,597,015; *n* = 4), 60,153,015 (±1,598,914; *n* = 4), and 68,500,895 (±2,535,550; *n* = 4) sequence reads were kept from red, yellow, and green individuals, respectively. Filtered reads mapped with similar rates of 84.01% (±0.95%), 84.2% (±0.69%), and 82.04% (±2.39%) for red, yellow and green individuals, respectively. These data are provided for each individual in Supplementary Materials Table S4. Transcriptome annotation is given in Supplementary Materials Table S5.

### 3.2. Transcriptome-Wide Functional Analysis

**Figure 2:**
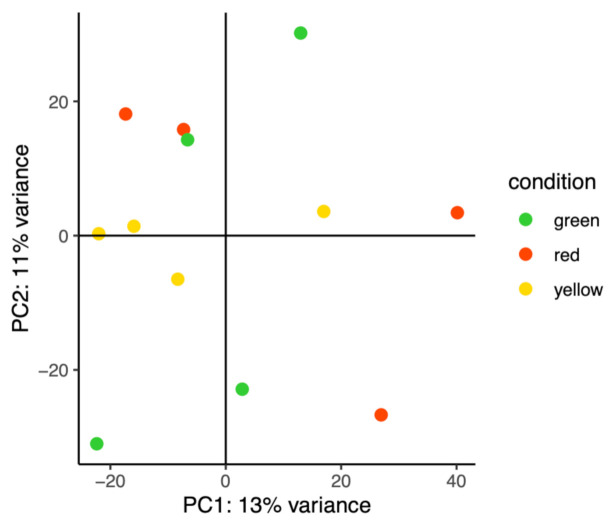
Principal component analysis (PCA) of genome-wide gene expression based on the negative binomial distribution of gene expression for all phenotypes. Each green, red or yellow dots represent an individual of the corresponding color.

PCA based on gene expression profiles of the 12 sequenced transcriptomes (4 individuals per color) shows that individuals are randomly distributed across the graph rather than clustered according to their color, suggesting that if transcriptomic regulation supports color phenotypes, it only involves a few genes and/or subtle differences in regulation. (Figure 2)

**Figure 3:**
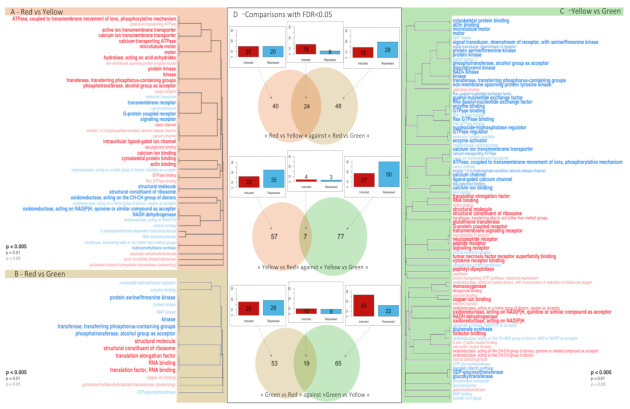
Enrichment analysis and differential expression. (**A**–**C**), Molecular functions significantly enriched by either over- (in red) or under- (in blue) expressed genes (**A**)—the red phenotype compared to the yellow, (**B**)—the red phenotype compared to the green, (**C**)—the yellow phenotype compared to the green. (**D**) Venn diagrams representing shared and specific genes differentially expressed (FDR < 0.05) between phenotypes. Histograms show the number of over- (red) or under-represented (blue) genes.

Three RBGOA analysis were performed: (i) red vs. yellow (Figure 3A), (ii) red vs. green (Figure 3B), and (iii) yellow vs. green (Figure 3C). Significantly enriched GO terms (*p* value < 0.01) were identified for each paired color combination: 45 GO terms for the comparison of red vs. yellow (27 over-represented and 18 under-represented), 16 GO terms for red vs. green, (7 over-represented and 9 under-represented), and 89 GO terms for yellow vs. green (36 over-represented and 53 under-represented). Among these enriched molecular functions, several belonged to pathways already known to be involved in pigmentation or biomineralization in various organisms [48,49,50]: “hydroxymethylbilane synthase activity” [48] (GO:0004418; under-represented in red vs. yellow), “oxidoreductases, acting on the CH-CH group of donors” [49,50] (GO:0016627; under-represented in red vs. yellow, but over-represented in yellow vs. green), “UDP-glycosyltransferase activity” [49] (GO:0008194; under-represented in yellow vs. green), and “UDP-glucosyltransferase activity” [49] (UGTs) (GO:0035251; under-represented in red vs. green and in yellow vs. green). The pterin and Raper–Mason pathways are also known to produce pigments [51,52,53], and molecular functions characteristic of these pathways were also significantly enriched. These included “GTPase activity” (GO:0003924) and “oxidoreductase, acting on NAD(P)H” (GO:0016651), both of which had a higher amount of transcripts in red vs. yellow and yellow vs. green; “monooxygenase activity” (GO:0004497), “tetrapyrrole binding” (GO:0046906), and “quinone binding” (GO:0048038), all over-represented in yellow vs. green; “phosphotransferase, alcohol group as acceptor” (GO:0016773), under-represented in yellow vs. green; “transferase activity, transferring alkyl or aryl (other than methyl) groups” (GO:0016765) and “glutathione transferase activity” (GO:0004364), both over-represented in yellow vs. green.

Two categories involved in purine metabolism, “oxidoreductase activity, acting on CH or CH2 groups” (GO:0016727) and “oxidoreductase activity” (GO:0016491) had lesser and greater amounts of transcripts in yellow vs. green, respectively. These categories included 21 genes coding for xanthine dehydrogenase, an enzyme known to degrade the yellow pigment xanthine into uric acid [51,54]. According to the literature and present knowledge, the other enriched GO terms identified in this study are not directly related to pigmentation or biomineralization but could still affect color expression directly or indirectly. These genes would make an interesting subject for future analyses.

### 3.3. DEG Functional Analysis

Differences in gene expression were analyzed by pairwise comparison of color phenotypes using DESeq2. In total, 71,059 genes were expressed with at least one read counted. Among these expressed genes, 64, 72 and 84 DEGs were identified (False Discovery Rate, FDR < 0.05) for the red vs. yellow, red vs. green, and yellow vs. green pairwise comparisons, respectively (DEGs for all pairwise comparisons are given in Supplementary Materials Tables S6–S8. With this approach, we hypothesized that a DEG present in two pairwise comparisons would be more likely to be associated with the phenotype present in both of these pairwise comparisons. We, therefore, drew Venn diagrams that identified 24 DEGs (18 over-represented, 6 under-represented) shared between the red vs. yellow and between red vs. green, 7 DEGs (4 over-represented 3 under-represented) shared between the red vs. yellow and yellow vs. green, and 19 DEGs (10 over-represented, 9 under-represented) shared between the red vs. green and yellow vs. green comparisons (Figure 3D). Among these DEGs, 16 red-associated, 3 yellow-associated, and 11 green-associated genes encode proteins of unknown function.

Several genes associated with the red phenotype are good candidates for explaining the red color. Among them, four genes encoding a porphobilinogen deaminase (PBGD) protein were under-represented (Log2FCs: (−2.91; −2.39) in red vs. green and Log2FCs: (−3.54; −2.77) in red vs. yellow). These genes had already been identified by the RBGOA analysis, and belong to “hydroxymethylbilane synthase activity” (GO:0004418). Porphobilinogen deaminase (KEGG entry K01749) can be involved in four pathways referenced in KEGG: “porphyrin and chlorophyll metabolism” (ko00860), “metabolic pathways” (ko01100), “biosynthesis of secondary metabolites” (ko01110), and “microbial metabolism in diverse environments” (ko1120). In addition, three DEGs belonging to the glycosyltransferase family were found under-represented in the red phenotype: (i) one glycosyltransferase family 8 protein, also found in the “transferase activity, transferring glycosyl groups” (GO:0016757; Log2FC: −2.63, for red vs. green and Log2FC: −2.61, for red vs. yellow); (ii) one glycosyltransferase-like protein LARGE also found in “transferase activity, transferring glycosyl groups” (GO:0016757) and “glucuronosyltransferase activity” (GO:0015020; Log2FC: −2.45 for red vs. green and Log2FC: −3.07 for red vs. yellow); and *iii*) one xylosyl- and glucuronyltransferase 1-like also found in the “transferase activity, transferring glycosyl groups” (GO:0016757), and “glucuronosyltransferase activity” (GO:0015020) molecular functions (Log2FC: −1.95 for red vs. green and Log2FC: −2.42 for red vs. yellow). The glycosyltransferase family 8 protein was found in “starch and sucrose metabolism” (tre00500). The glycosyltransferase-like protein LARGE and xylosyl- and glucuronyltransferase 1-like could both be involved in two pathways: “mannose type O-glycan biosynthesis” (ko00515) and “metabolic pathways” (ko01100). Finally, the under-represented decaprenyl-diphosphate synthase subunit 2-like was also found in the “transferase activity” (GO:0016740) molecular function by RBGOA (Log2FC: −2.88 for red vs. green and Log2FC: 3.006 for red vs. yellow). The “terpenoid backbone biosynthesis pathway” (ec00900) was found to be associated with the red phenotype. Finally, although we cannot exclude their involvement in color expression, none of the yellow- and green-associated DEGs had sufficient background literature or sequence similarity to a gene of known function for us to draw a direct link to pigmentation.

Among the DEGs present in only one pairwise comparison, we identified four interesting candidate genes linked to the green phenotype. The first two of these candidates, which were found under-represented in the green vs. yellow comparison and encode two glutathione-S-transferase (GST) enzymes (Log2FC: −1.02 and −1.27), were also found in the “glutathione transferase activity” (GO:0004364) molecular function in RBGOA and are involved in “glutathione metabolism” (ec00480), “metabolism of xenobiotics by cytochrome P450” (ec00980), and in “drug metabolism–cytochrome P450” (ec00982). Interestingly, GST has been shown to balance the production of eumelanin and pheomelanin [55]. The two other candidates encoded transcobalamin-2 found in the “copper ion binding” (GO:0005507) molecular function in RBGOA and were present in lesser quantities in green vs. red (Log2FC: −3.02) and green vs. yellow (Log2FC: −2.70). According to KEGG classification, transcobalamin-2 (K14619) is involved in the vitamin digestion and absorption pathway (mmu04977). Interestingly, vitamins such as the B12 (cobalamin) could also be related to pigment synthesis. Detailed information on DEGs is given in Supplementary Materials Tables S6–S8.

### 3.4. Enzymatic Structure Analysis with Homology Modeling

In our DEGs results, four genes encoding for a porphobilinogen deaminase (PBGD) were found in the red-associated analysis and this gene is known to be involved in red coloration in some Mollusca [56]. Since we have very strong evidence for the involvement of PBGDs in the expression of red color, we checked whether these enzymes displayed sufficient structural and biochemical similarities with a reference PBGD to be considered active. We did so by comparing their primary, secondary and tertiary structure to human PBGD, which has been studied by crystallography (chain A of the 3ECR) [42]. K98, D99, R149, R150, R173, R195, Q217, and C261 are key residues for catalysis with the cofactor [42]; S96, H120, and L238 enable the movement of the protein domains; and R116, R225, D228, and L278 ensure the stability of movement. R167 is involved in the release of the final product [42].

**Figure 4:**
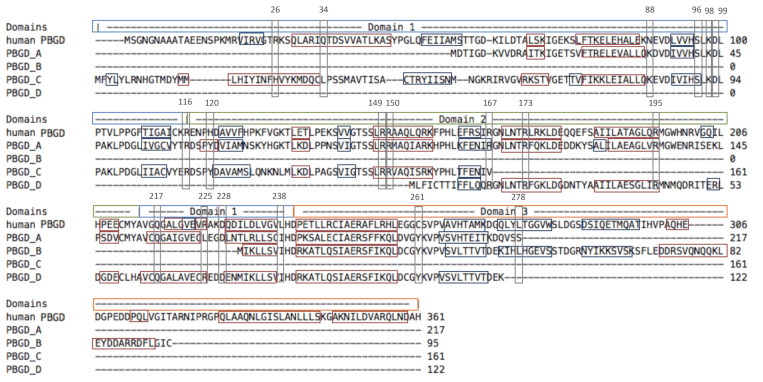
Alignments of the four *P. margaritifera* porphobilinogen deaminase (PBGD) (PBGD_A to PBGD_D) against the human PBGD (Song et al. 2009). The pale blue boxes correspond to domain 1, the green to domain 2 and the orange to domain 3. Red boxes show α helix (H) and dark blue boxes β strands (S). Grey boxes correspond to key residues (amino acids).

At the primary structure level, the sequences were 306, 217, 95, 161, and 122 amino acids long for human PBGD and the four *P. margaritifera* PBGD (A to D), respectively (Figure 4). *P. margaritifera* PBGD_A to _D displayed 54.3%, 30.3%, 37.4%, and 42.6% of similarity with human PBGD, respectively. Alignment with human PBGD produced scores of 273, 69, 120, and 122 and coverage of 66%, 21%, 32%, and 33% (*e*-value 5e-91, 2e-14, 2e-32, and 2e-33) for PBGD_A to D, respectively. Based on these results, the four *P. margaritifera* PBGD can be considered as homologous to human PBGD [57].

**Figure 5:**
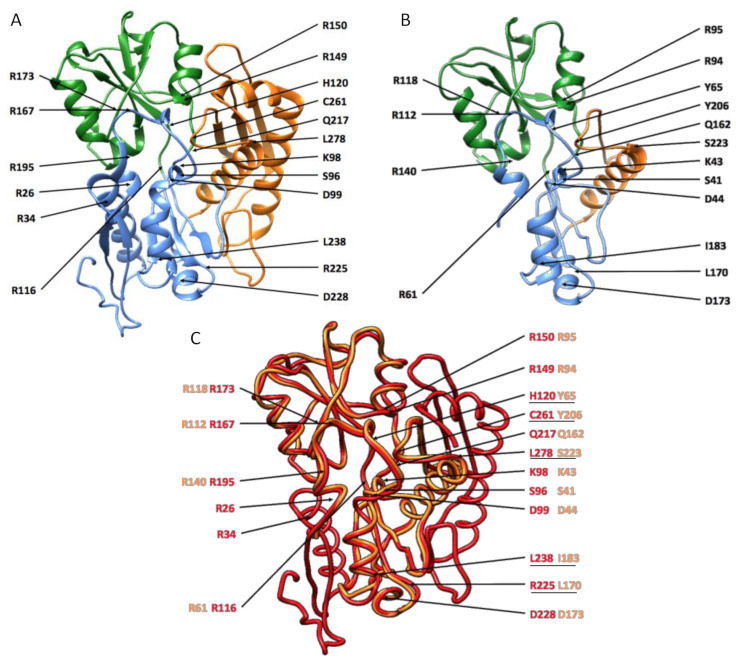
Enzymatic structure and homology modeling for human PBGD (**A**) and *P. margaritifera* PBDG_A (**B**). Domain one is shown in blue, domain two in green, and domain three in orange. Key residues are labeled, as are their positions in the primary structure. (**C**) Superimposition of *P. margaritifera* PBGD_A (in orange) on human PBGD (in red). Positions of key residues for each enzyme are indicated, and amino acid substitutions are underlined.

At the secondary and tertiary structure levels, the succession of α helix and β strands is mainly conserved, although some differences can be observed (Figure 4). Most importantly, the key amino acids for enzymatic processing (Figure 4, Table 1) were retrieved in the *P. margaritifera* PBGDs, especially isoform A, which appeared to be the most conserved. In this PBGD_A, the binding and the interaction with the dipyrromethane cofactor may be performed by residues K43, D44, R94, R95, R118, R140, Q162, and Y206 (Figure 4 and Figure 5, Table 1). Hinge residues that enable the movement (S41, Y65, and I183) and stability (R61, L170, D173, and S223) of the protein domains were also identified (Figure 4 and Figure 5, Table 1). Finally, R112, an essential residue for the release of the substrate was also present (Figure 4 and Figure 5, Table 1). In addition to this biochemical conservation, these key residues occupy a similar position in PBGD_A and human PBGD (Figure 5).

Overall, the enzymatic structure analyses demonstrated conserved general biochemical and structural properties of protein domains, despite variations in some amino acid sequences. We can therefore hypothesize that PBGD_A is active in *P. margaritifera*, which would be expected since even PBGDs displaying high structural variation have been reported several times as being active [42,58,59,60].

### 3.5. Raman Spectra Analysis

**Figure 6:**
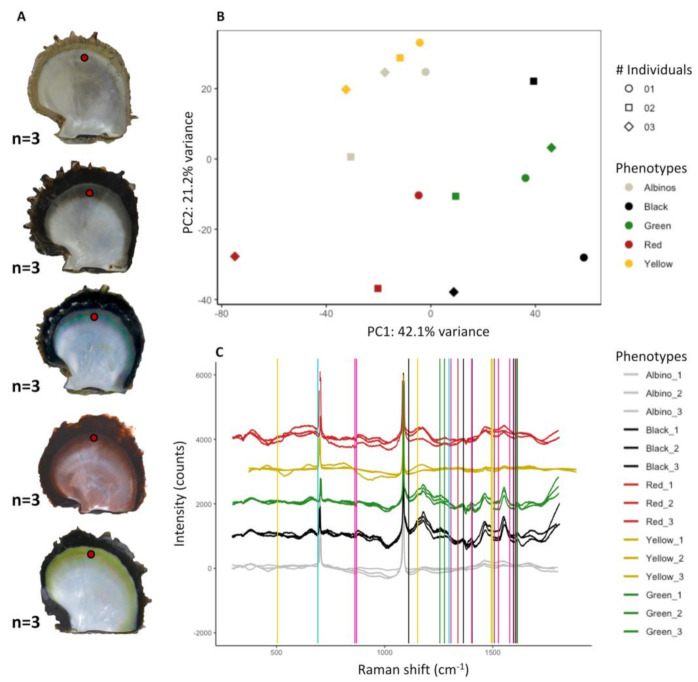
Raman spectroscopy results. (**A**): Experimental design of RAMAN validation. Three individuals per phenotype were studied. Red dots show s where on the inner shell Raman spectra were produced. (**B**): Principal component analysis of raw Raman spectra obtained for the 5 inner shell colors analyzed (3 individuals per color). (**C**): calibrated spectra presenting the intensity (counts) of the signal as a function of the Raman shift (cm^−1^). Colored vertical lines mark the positions of peaks constituting a Raman signature specific to one of the identified pigments: forest green, biliverdin; dark sea green, cobalamin; red, uroporphyrin I; dark turquoise, bilirubin; gold, phaeomelanin; black, melanin; and deep pink, xanthine. Enlargements of the spectra for each signature are provided in the Supplementary Materials Figure S1.

The transcriptomic approach identified molecular pathways that were differentially regulated between phenotypes. To test the involvement of these pathways in the expression of the red, yellow, or green colors, we performed Raman spectra analysis on five different phenotypes: the red, yellow, and green. Albino (no color) and black (in which a mix of colors are expressed to a lesser degree among different individuals) phenotypes were used as controls. PCA analysis performed on raw Raman spectra (Figure 6) reveals two distinct clusters, one including the yellow and albino phenotypes and the red, the second including black and green phenotypes. In this last cluster, the variability in Raman spectra is lower for the green phenotypes, for which individuals grouped together more closely, compared with the black and red, which show a wider distribution (Figure 6).

Individual spectra display the characteristic peaks of high intensity corresponding to the CaCO_3_ biomineral in its aragonitic and calcitic forms. This high intensity reflects that the shell is essentially composed of these two polymorphs [61]. At a lower intensity, dozens of peaks are present and illustrate the high chemical complexity of this biomaterial, with more than 78 different Shell Matrix Proteins (90% of the organic matrix [61,62]), at least 4 different lipid’s family (fatty acids, cholesterol, phytadienes, and ketones; 0.15 to 0.29% of the organic matrix [61]); 10 different traces of heavy metals [63], polysaccharides, and secondary metabolites [61].

Among the 174 peaks included in our homemade database (see Supplementary Materials Table S3), 40 peaks were found in the spectra obtained from our red, yellow, and green individuals (Table 2 and Supplementary Materials Figure S1). The combination of these peaks represents the signatures corresponding to nine pigments previously proposed by the transcriptomic approach: uroporphyrin I or III (three peaks), copper-uroporphyrin (six peaks), FeIII-uroporphyrin (six peaks), biliverdin (four peaks), cobalamin (two peaks), pheomelanin (four peaks), eumelanin (five peaks), xanthine (eight peaks), and bilirubin (two peaks). The complete set of peaks characteristic of each molecule were not identified and/or were not present in all individuals of the same phenotype. This phenomenon is classically observed in Raman spectroscopy, where the absence of a peak does not demonstrate the absence of a molecule, whereas the presence of a peak can be considered as evidence, a phenomenon also reported for pure pigments such as the pheomelanin [64].

Likewise, most of the pigments corresponding to a specific color displayed some of their signature peaks in individuals of different phenotype, but with variation in their intensity (Table 2). These results are validated by the absence of detection of peaks characteristic of each pigments in the albino control, a phenotype displaying an absence of coloration.

Red phenotypes were mainly characterized by peaks corresponding to uroporphyrin I or III or its derivatives, copper or FeIII-uroporphyrin (Table 2). Red individuals also displayed Raman signatures characteristic of the green cobalamin, yellow xanthine, and black eumelanin, although this non-red signature was shown to a lesser degree (Table 2, Supplementary Materials Table S9). None of these peaks were retrieved in the albino control.

Green phenotypes were mainly characterized by peaks corresponding to the green pigments biliverdin and cobalamin (Table 2, Supplementary Materials Table S9). Green phenotypes also had a strong signal for the yellow pigment xanthine and the black pigment eumelanin (Table 2). Signatures of uroporphyrin I (red) and bilirubin (yellow) were also present but minor. These results, showing a large diversity of pigments in the green phenotypes, confirm that it is the most complex. None of these peaks were retrieved in the albino control.

Yellow phenotypes were mainly characterized by the two yellow pigments pheomelanin and bilirubin. At a lesser proportion, they also showed signatures characteristic of xanthine (yellow pigments), uroporphyrin I (red), and eumelanin (black), also in minor proportions. None of these peaks were retrieved in the albino control.

The black and albino phenotypes had served as controls. As expected, the black phenotype did not have a signature for one particular color and presented an average proportion of all the identified pigments (except green). The albino did not show any peaks, which confirmed the absence of pigmentation from these samples.

## 4. Discussion

*P. margaritifera* is known for its ability to express the broadest range of internal shell and pearl colors of all pearl oyster species [65]. To characterize pathways and pigments underlying these colors, we developed an integrated approach combining experimental strategy to minimize the impact of phenotypic diversity and environmental effects, genome-wide transcriptomics, and Raman spectroscopy. The combination of these two last methods has first revealed that monochrome shell does not exist in this species, except in the particular case of albino. Indeed, all the identified pathways and pigments are transcribed ubiquitously, and the Raman signatures of red, yellow, and green pigments were retrieved in varying proportions in all phenotypes (except albino). The dominant color of an inner shell is, therefore, due to the preferential expression of one pathway relative to another. Second, the analyses revealed the central role played by the heme pathway, involved at different levels in the production of red (uroporphyrin and derivates), yellow (bilirubin), and green (biliverdin and forms of cobalamin) pigments. Additionally, but to a lesser extent, the Raper–Mason, and purine metabolism pathways were also shown to produce the yellow pigments pheomelanin and xanthine and the black pigment eumelanin.

### 4.1. The Inner Shell of P. margaritifera: Chemically Complex, Polychromic, but Containing Dominant Pigments

Mollusca shell is a biomaterial constituted by two distinct types of component—the mineral and the organic layer. The former has a composition of CaCO_3_ that can take different form (aragonite, calcite etc.). The organic layer is much more complex and harbor dozens of different proteins, polysaccharides, lipids, traces of metals, and secondary metabolite [61,62]. This cocktail of molecules forms a highly complex assemblage where only a very small fraction will determine the inner shell pigmentation, making it a challenge to detect them. This challenge is even increased by the diffuse and unstable nature of the inner shell color of *P. margaritifera* [66,67], which all together explains the complexity and the low intensity of the Raman spectra we obtained, highlighting the need to combine several methods to tackle the same question from different angles. This combined approach revealed differences in gene expression levels between phenotypes rather than a binary pattern of expression (expressed/unexpressed), highlighting the polychromic nature of the inner shell. This was further confirmed by the Raman spectra, bringing another set of evidence by the detection of the same pigments in various phenotypes, but with different intensities. This concomitant analysis raises the conclusion that all oysters express all pigments but in various proportions, which gives rise to the dominant color. This is further confirmed by the absence of Raman’s signature for the unpigmented albino specimens, but also visually (Figure 1) where a reddish band can be observed on the green shell and a greenish and reddish band on the yellow shell. This polychromic nature of the black-lipped pearl oyster is an original characteristic of the species and is incidentally exploited in pearl farming [65].

### 4.2. Dysfunctions of Porphobilinogen Deaminase in the Heme Pathways Produce Red Uroporphyrin and its Derivates in Red Individuals

**Figure 7:**
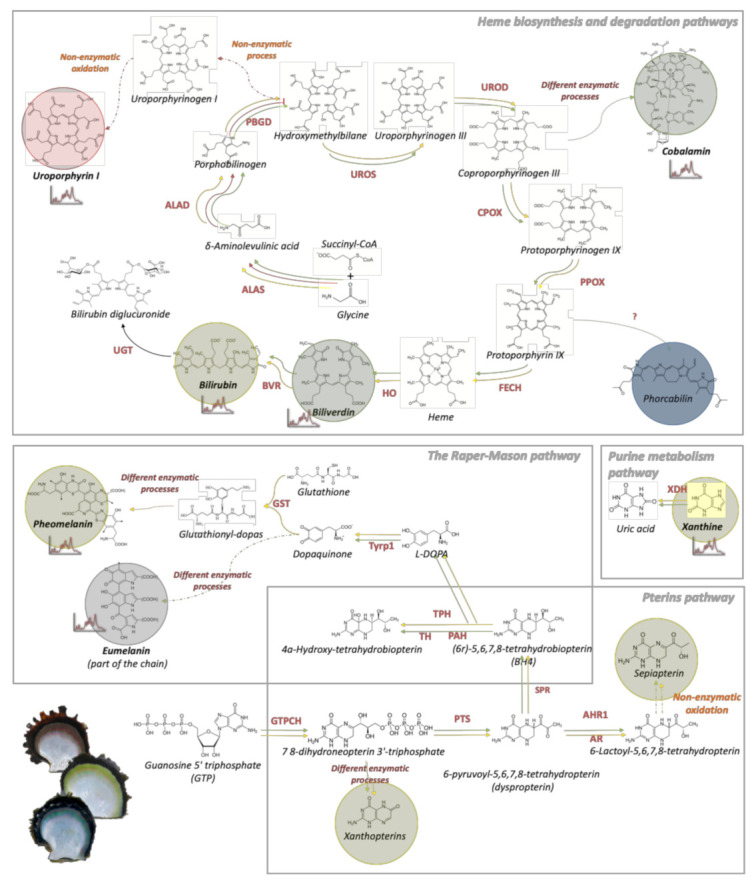
The four identified pathways responsible for *P. margaritifera* inner shell color: the heme biosynthesis and degradation pathways, the pterins pathway, the Raper–Mason pathway, and the purine metabolism pathway. The putative pigments are shown with colored circles; those validated by Raman spectroscopy have a Raman graph symbol. The red, yellow, and green arrows refer to the synthesis of red, yellow, and green pigments, respectively. ALAS: aminolevulinic acid synthase; ALAD: delta-aminolevulinic acid dehydratase; PBGD: porphibilinogen deaminase; UROS: uroporphyrinogen III synthase; UROD: uroporphyrinogen decarboxylase; CPOX: coproporphyrinogen oxidase; PPOX: protoporphyrinogen oxidase; FECH: ferrochelatase; HO: heme oxygenase; BVR: biliverdin reductase; UGT: UDP-glucuronyltransferase; GTPH: GTP cyclohydrolyase I; PTS: 6-pyruvoyltetrahydropterin/6-carboxytetrahydropterin synthase; AR: aldose reductase; AHR1: aldehyde reductase 1; SPR: sepiapterin reductase; PAH: phenylalanine-4-hydroxylase; TPH: tryptophan 5-monooxygenase; TH: tyrosine 3-monooxygenase; Tyrp1: tyrosinase-related protein 1; GST: gluthathione-S-transferase; XDH: xanthine dehydrogenase.

Among the candidate pathways identified, the heme biosynthesis and degradation pathways appear to be central (Figure 7). Hemes are most commonly recognized as components of hemoproteins such as hemoglobin, cytochromes, or catalases [68] and are excreted after their degradation [68]. We identified four DEGs encoding a porphobilinogen deaminase (PBGD), one of the main enzymes in the heme pathway. All of these genes were under-represented in the red phenotype according to the DEG functional analysis. Homology modeling illustrates that at least PBGD_A is functional since it displays the key biochemical and structural characteristics needed to catalyze its enzymatic reaction (Figure 5). PBGD is the third enzyme of the heme pathway and catalyzes the production of hydroxymethylbilane from four molecules of porphobilinogen in the presence of dipyrromethane as a cofactor [69]. In humans, deficiency of PBGD is characteristic of Acute Intermittent Porphyria (AIP), a genetic disease in which the main symptom is red urine coloration due to uroporphyrin overproduction [70,71]. The molecules of hydroxymethylbilane that are produced are converted to uroporphyrinogen I and then to uroporphyrin I by a non-enzymatic process and oxidation (Figure 7), respectively [48,71,72]. Uroporphyrin I is a well-known pigment in Mollusca: it has been previously identified by spectrophotometry and HPLC techniques in yellow-brown snail [2,48] and in the inner and outer shells and cultured pearls of *P. margaritifera* [18,19]. The color variation of the shells of *P. margaritifera* may be associated to variation in the concentration of the pigments, or more probably, to the proportion of the different derivate of uroporphyrin that are associated with metal ion [48,73]. Our Raman spectroscopy analysis strengthens this last hypothesis since it highlights the presence of derivatives of uroporphyrin: copper and FeIII uroporphyrin. Because of its toxicity, uroporphyrin I is usually excreted, which is mainly achieved through urine in mammals [74] and, putatively, through deposition in the shell in Mollusca (the shell is well known to bio-accumulate toxic compounds in Mollusca [75]). In this context and based on actual knowledge, we propose that the red color in *P. margaritifera* originates from deregulation of the heme pathway at the level of PBDG. Interestingly, the crossing of red individuals produces a dominant red F1 [24], which argues in favor of an oligogenic basis for the red phenotype as is the case for human AIP [76]. This overproduction of uroporphyrin does not appear to be detrimental for the pearl oysters since this phenotype occurs naturally in wild populations, sometimes at a non-negligible frequency [65].

### 4.3. Heme, Raper–Mason, and Purine Metabolism Pathways Produce Bilirubin, Pheomelanin, and Xanthine Pigments Underlying the Yellow Phenotype

The first yellow pigment identified in the present analysis, bilirubin, was also related to the heme pathway. In the downstream part of the heme degradation pathway, heme oxygenase (HO) converts heme into biliverdin [77], which is then transformed into bilirubin (a well-known yellow pigment in animals [77,78,79]) through the action of biliverdin reductase (BVR; Figure 7). Although HO and BVR genes were already found expressed in Mollusca species [56,80], these two genes were not found in our analysis. However, the gene encoding a UDP-glucuronosyltransferase (UGT), the enzyme that degrades bilirubin into bilirubin diglucuronide [81,82] (a colorless compound) was found under-represented in the yellow phenotype compared with the green and red, suggesting a possible accumulation of bilirubin in the animal, later excreted and deposited into the shell. In addition, other genes belonging to the UGT family were found in high numbers in the GO categories “UDP-glycosyltransferase” and “UDP-glucosyltransferase”, two molecular functions expressed at a lesser degree in the yellow compared with the green phenotype. The involvement of the bilirubin in the yellow phenotype was also confirmed by Raman spectroscopy results, showing specific signatures of the bilirubin mainly in the yellow individuals. The bilirubin is a well-known pigment in vertebrates but was not yet detected in invertebrates. Our study provides a first line of evidence of its synthesis in invertebrates as well, but this should be further confirmed by additional analyses enabling the chemical purification of this compound to provide a definitive confirmation.

The second identified pathway that may lead to the production of yellow pigment was the purine metabolism pathway, ending with the production of xanthine (Figure 7). RBGOA analysis revealed the over-representation of categories that contain at least 21 genes coding for xanthine dehydrogenase, an enzyme known to degrade the yellow pigment xanthine into uric acid [51,54]. Some of these 21 genes were more expressed in the yellow phenotype, while others were more expressed in the green phenotype, suggesting a generalized production of this pigment. Raman spectroscopy revealed the specific signatures of 7/16 referential peaks of xanthine in all phenotypes except in albino (0/16). All these results strengthen the possibility that xanthine synthesis through the purine metabolism pathway is involved in the expression of the yellow phenotype.

The third identified pathway leading to the production of yellow pigments for which we obtained transcriptomic and Raman evidence is the Raper-Mason pathway that leads to the yellow pheomelanin [53]. The Raper–Mason pathway starts with the L-DOPA, which is transformed into dopaquinone by the activity of tyrosinase-related protein 1 (Tyrp1). Dopaquinone could either become the black pigment eumelanin [83], through a non-enzymatic process, or the yellow pigment pheomelanin [83] by the successive action of glutathione-S-transferase (GST) and glutamine γ-glutamyltransferase [55]. Both of these pigments have already been described in Mollusca [84]. The molecular function “glutathione transferase activity” (GO:0004364), as well as two GST genes, were over-represented in the yellow phenotype (relative to the green phenotype). Since the ratio between the production of pheomelanin and eumelanin is a fine balance regulated by GST [55] activity, it is probable that yellow shells are enriched in yellow pheomelanin in comparison to green ones. This hypothesis was validated by the Raman spectroscopy results confirming the presence of the 4/4 specific peaks for the pheomelanin only in the yellow individuals and 2/4 in some black individuals. In addition, Raman spectroscopy results also indicated the presence of black eumelanin with a strong degree of confidence in the green phenotype (several signatures), while this was almost undetected in the yellow shells. This result confirms the proposed regulation pathway whereby the production of pheomelanin or eumelanin is controlled by fine-tuning regulation of GST activity.

In summary, we can conclude that the yellow phenotype results from the production and accumulation of several pigments, mainly bilirubin, xanthine, and pheomelanin (Figure 7). These three pigments come from three different pathways, which increase the potential for high phenotypic variation among individuals of this color phenotype but also of the others. Indeed, it is not rare to observe shades of yellow in green, black, or even red shells as well as different sheds of yellow among yellow individuals [65,83].

### 4.4. Heme Pathways Are Central to the Green Phenotype through the Production of Biliverdin and Green Forms of Cobalamin

Green pigments are uncommon in animals [84,85] and usually originate from three non-exclusive sources: a mixture of black and yellow pigments, a mixture of blue and yellow pigments, or, more rarely, from an actual green pigment [86]. The mixture of black and yellow pigments to create a green color is interesting in our case, since the yellow and green phenotypes appear to be transcriptionally very similar to each other. The green pearl oyster phenotype could result from the mixture of yellow pigments (i.e., Pheomelanin, xanthine and bilirubin) with the black pigment eumelanin produced by the Raper–Mason pathway as described above.

The mixture of blue and yellow pigments is also a plausible scenario. Although blue color phenotypes are rare in pearl oyster and no blue pigment has been clearly identified to date, *P. margaritifera* is known to produce blue internal shells and pearls. In some butterflies, the blue pigment phorcabilin [87,88] is mixed with yellow pigments to produce a green color [87,88]. The biosynthesis of phorcabilin takes place in the heme biosynthesis pathway, resulting from the oxidation of protoporphyrin IX into pterobilin, followed by its conversion into phorcabilin by a non-enzymatic process [88]. This pathway remains underexplored: several of its steps are not completely understood, and the oxygenase catalyzing the conversion of protoporphyrin IX in pterobilin is still unknown. However, in our RBGOA comparison of yellow and green phenotypes, some oxygenase-like genes were identified and observed to be over-represented in the green phenotype. Since no reference Raman signatures of phorcabilin are yet available, we were not able to either confirm or deny its presence in the green shells. Validation of this hypothesis will therefore require further experiments.

Considering the third possible source of green color, the direct synthesis of a green pigment, biliverdin, is a good candidate. This pigment has already been identified in Mollusca [89] and is produced in the heme degradation pathway [90]. Although we did not identify genes encoding the heme oxygenase and biliverdin reductase enzymes that are necessary for the production and the degradation of biliverdin, our Raman spectroscopy results showed its presence in the green shells. Since the heme pathway is well conserved in the animal kingdom [56], the absence of these genes in *P. margaritifera* is unlikely and is here probably due to assembly or annotation errors in the reference genome. The heme degradation pathway can also produce another green pigment, cobalamin. Cobalamin is a hemic molecule derived from coproporphyrinogen III [91] and some of its forms can be green [73,91,92]. Another interesting feature of cobalamin is its bacterial-dependent synthesis [93,94,95] that often relies on a symbiotic interaction between a host and bacteria [96,97,98,99]. Alternatively, cobalamin can be directly acquired by food uptake [96]. In our data, the next two enzymes after coproporphyrinogen III (the cobalamin precursor), coproporphyrinogen oxidase (CPOX), and protoporphyrinogen oxidase (PPOX), were under-expressed in the green phenotype than the yellow according to our RBGOA results. In addition, two genes encoding a transcobalamin-2 were significantly under-expressed in the green phenotype compared with the yellow or red phenotypes. Transcobalamins are proteins involved in the transport and excretion of cobalamins [100]. This transcriptomic evidence is strengthened by the Raman spectroscopy results which indicated the presence of specific signatures of cobalamin in green individuals. At the species level, this result suggests symbiotic interaction between the oyster and some of the bacteria composing their microbiome. This bacterium would be involved in the production of cobalamin, and the green phenotype would be due to a less efficient transport and excretion of this pigment in comparison to other phenotypes.

In summary, the green phenotype in pearl oysters appears to arise in two different ways: the direct production of green pigments biliverdin and green cobalamin forms by the heme pathway, and the indirect production of a green color through the mixing of black eumelanin and yellow pigments from the Raper–Mason pathway.

## 5. Conclusions

To conclude, we demonstrated that the heme pathways appear to be central in the inner shell coloration of *P. margaritifera*. The red phenotype is clearly linked to the reduced expression of an enzyme, PBGD, likely originating from a genetic mutation. The yellow and green phenotypes appear to be more interlinked and share several pathways. Interestingly, the Raman spectra obtained highlighted that any of the studied phenotypes could be characterized as pure or monochromatic even among populations produced to be monochromatic such as the three populations used in this study. This indicates that all these pathways and pigments are probably expressed universally in all individuals and that the dominant color comes from gene expression variations between individuals, a phenomenon that can probably be extended to many other organisms with large chromatic variation.

In the wild, the inner shell color of most *P. margaritifera* individuals is either grey/black or is highly polychromic. This characteristic leads to a high level of uncertainty in the future color of the pearls produced by the farming process based on wild-collected pearl oysters [65]. Producing pearls with saturated monochromatic colors is a promising way to “produce fewer, but better” pearls [22]. Our results show that reaching this goal through selective breeding will be challenging given the natural tendency toward polychromy and the diversity of pigments, genes, and pathways involved in this phenotypic trait. However, this work constitutes a significant step in the understanding of the molecular mechanisms underlying the color of *P. margaritifera* and pearls produced through its culture. This work also built solid foundations for the development of future research that will contribute to a sustainable pearl industry. Indeed, our results will be used to develop marker-assisted selection [101,102] to strengthen genetic selection programs for elite donor pearl oysters [25].

## Figures and Tables

**Table 1:** Conservation of key residues between *P. margaritifera* PBGDs and human PBGD. Amino acid numbering is based on the alignment given in Figure 5.

Human’s_PBGD	PBGD_A	PBGD_B	PBGD_C	PBGD_D
R26	NA	NA	H23	NA
Q34	NA	NA	L32	NA
N88	K33	NA	K82	NA
S96	S41	NA	S90	NA
K98	K43	NA	K92	NA
D99	D44	NA	D93	NA
R116	R61	NA	R110	NA
H120	Y65	NA	Y114	NA
R149	R94	NA	R143	NA
R150	R95	NA	R144	NA
R167	R112	NA	V161	R14
R173	R118	NA	NA	R20
R195	R140	NA	NA	R42
Q217	Q162	NA	NA	Q64
R225	L170	NA	NA	R72
D228	D173	NA	NA	D75
L238	I183	I8	NA	Y85
C261	Y206	Y31	NA	Y108
L278	S223	L48	NA	NA

**Table 2:** Pigment validation by Raman spectroscopy, synthetic results. Raman signatures characteristic of each pigment found in the literature; Peak characterization (identify peaks matching or not expected signals), peak intensity (the value of the lowest point of the peak minus the value of the peak) and ratio relative to the intensity of the calcite reference peak (ratio was calculated between the intensity of the selected peak (calculated as previously described) and the peak of the referential calcite for each spectrum). Each red, yellow, green, black or white background/text color are corresponding to the phenotype color. The full table is presented in Supplementary Materials Table S9.

Pigment	Peaks	Publication	Red individual 1			Red individual 2			Red individual 3			Yellow individual 1			Yellow individual 2			Yellow individual 3			Green individual 1			Green individual 2			Green individual 3			Black individual 1			Black individual 2			Black individual 3			Albino individual 1			Albino individual 2			Albino individual 3		
			Peak	Intensity	Ratio	Peak	Intensity	Ratio	Peak	Intensity	Ratio	Peak	Intensity	Ratio	Peak	Intensity	Ratio	Peak	Intensity	Ratio	Peak	Intensity	Ratio	Peak	Intensity	Ratio	Peak	Intensity	Ratio	Peak	Intensity	Ratio	Peak	Intensity	Ratio	Peak	Intensity	Ratio	Peak	Intensity	Ratio	Peak	Intensity	Ratio	Peak	Intensity	Ratio
**Uropor--phyrin I**	**1335**	Williams et al. 2017	**yes**	**29.26**	**0.66**	**yes**	**19.70**	**0.49**																**yes**	**16.05**	**0.50**																					
	**1445**		**yes**	**44.39**	**0.83**	**yes**	**29.10**	**0.72**													**yes**	**120.49**	**0.77**				**yes**	**46.51**	**0.64**																		
	**1556**														**yes**	**13.18**	**0.57**																														
**Copperuroporphyrin**	**1310**	Shelnutt 1981, 1982, et al. 1984	**yes**	**6.52**	**0.66**	**yes**	**5.63**	**0.46**	**yes**	**5.37**	**0.22**																																				
	**1379**								**yes**	**35.73**	**0.25**																																				
	**1403**								**yes**	**11.34**	**0.27**																			**yes**	**106.76**	**0.51**	**yes**	**99.57**	**0.41**	**yes**	**62.13**	**0.43**									
	**1499**		**yes**	**27.21**	**0.73**	**yes**	**19.07**	**0.74**	**yes**	**8.45**	**0.31**																																				
	**1582**					**yes**	**17.18**	**0.71**																																							
	**1637**					**yes**	**10.28**	**0.66**																																							
**FeIIIuroporphyrin**	**1307**	Shelnutt 1982	**yes**	**5.48**	**0.66**	**yes**	**11.81**	**0.45**																																							
	**1376**		**yes**	**9.29**	**0.62**	**yes**	**29.43**	**0.51**	**yes**	**21.71**	**0.23**																**yes**	**59.22**	**0.49**																		
	**1402**		**yes**	**11.29**	**0.67**	**yes**	**16.37**	**0.54**	**yes**	**11.34**	**0.26**																			**yes**	**114.98**	**0.51**															
	**1489**		**yes**	**12.80**	**0.76**	**yes**	**16.93**	**0.74**	**yes**	**8.04**	**0.31**																			**yes**	**111.01**	**0.73**	**yes**	**42.97**	**0.51**												
	**1581**		**yes**	**15.64**	**0.70**				**yes**	**4.23**	**0.30**																																				
	**1627**		**yes**	**16.99**	**0.72**																																										
**Biliverdin**	**1252**	Margulies and Toporowicz 1984, Holt et al. 1989																									**yes**	**40.58**	**0.81**																		
	**1273**																							**yes**	**24.18**	**0.56**																					
	**1606**																				**yes**	**11.07**	**0.73**	**yes**	**20.05**	**0.56**	**yes**	**12.97**	**0.60**																		
	**1608**																				**yes**	**38.36**	**0.73**	**yes**	**7.60**	**0.56**	**yes**	**21.08**	**0.60**																		
**Cobala--min**	**1495**	Puckett et al 1996, Galluzzi et al. 1974																			**yes**	**16.70**	**0.72**	**yes**	**14.17**	**0.57**																					
	**1502**					**yes**	**19.50**	**0.75**													**yes**	**18.87**	**0.72**	**yes**	**15.99**	**0.56**	**yes**	**24.57**	**0.53**																		
**Xanthine**	**862**	Muniz-Miranda et al. 2018																**yes**	**25.06**	**0.48**	**yes**	**29.51**	**0.71**				**yes**	**17.56**	**0.60**																		
	**870**		**yes**	**79.86**	**0.71**	**yes**	**35.32**	**0.57**	**yes**	**81.36**	**0.21**										**yes**	**30.95**	**0.72**	**yes**	**38.01**	**0.53**	**yes**	**111.64**	**0.66**							**yes**	**45.10**	**0.51**									
	**1303**											**yes**	**7.64**	**0.54**	**yes**	**9.09**	**0.53**																														
	**1398**																				**yes**	**5.58**	**0.68**	**yes**	**2.73**	**0.57**	**yes**	**7.76**	**0.55**																		
	**1521**					**yes**	**25.67**	**0.75**										**yes**	**8.88**	**0.48**							**yes**	**41.97**	**0.51**																		
	**1573**																							**yes**	**28.38**	**0.56**	**yes**	**6.44**	**0.55**	**yes**	**42.71**	**0.60**															
	**1593**																																														
	**1598**																	**yes**	**9.26**	**0.53**																											
**Pheomelanin**	**507**	Galvan et al. 2013										**yes**	**3.67**	**0.54**				**yes**	**26.49**	**0.48**																											
	**1150**																	**yes**	**17.71**	**0.56**										**yes**	**42.43**	**1.00**															
	**1488**											**yes**	**2.08**	**0.53**	**yes**	**2.69**	**0.55**	**yes**	**0.43**	**0.48**																											
	**1490**											**yes**	**1.11**	**0.53**	**yes**	**1.92**	**0.55**	**yes**	**1.48**	**0.47**										**yes**	**92.35**	**0.72**				**yes**	**70.77**	**0.63**									
**Bilirubin**	**692**	Dybas et al. 2018										**yes**	**22.07**	**0.55**	**yes**	**13.70**	**0.56**																														
	**1294**														**yes**	**5.46**	**0.53**	**yes**	**4.40**	**0.51**	**yes**	**32.05**	**0.70**				**yes**	**23.52**	**0.66**																		
**Melanin**	**1109**	Centeno and Shamir 2008, Williams et al. 2016				**yes**	**27.08**	**0.71**	**yes**	**34.65**	**0.24**										**yes**	**20.82**	**0.79**																								
	**1360**																	**yes**	**12.87**	**0.49**	**yes**	**12.00**	**0.67**																								
	**1400**																				**yes**	**71.58**	**0.67**	**yes**	**54.95**	**0.57**	**yes**	**27.01**	**0.54**				**yes**	**39.33**	**0.43**	**yes**	**44.05**	**0.46**									
	**1590**																							**yes**	**13.25**	**0.52**																					
	**1600**																							**yes**	**23.14**	**0.53**																					

## Data Availability

Data can be found in Supplementary Materials section.

## Supplementary Materials

The following are available online at https://www.mdpi.com/2073-4425/12/3/421/s1, Figure S1: Raman spectra and enlargement corresponding to Raman signatures of interest, Table S1: Detailed parameters of the bioinformatic pipelines, Table S2: Detailed protocol of the homology modeling, Table S3: Homemade bibliographic database referencing Raman peaks specific to pigments, Table S4: a) Detailed results of FastQC for each individual (R1 = Reverse & R2 = Forward) before and after Trimmomatic (X16–X19 = red individuals; X21–X25 = yellow individuals; X26–X30 = green individuals). b) FlagStat results for the mapping of each individual (X16–X19 = red individuals; X21–X25 = yellow individuals; X26–X30 = green individuals), Table S5: Transcriptome annotation, Table S6: DEGs and corresponding BlastX results for the comparison “Red versus Yellow”. The colored cells correspond to the color-specific DEGs that can be found in two comparisons, Table S7: DEGs and corresponding BlastX results for the comparison “Red versus Green”. The colored cells correspond to the color-specific DEGs that can be found in two comparisons, Table S8: DEGs and corresponding BlastX results for the comparison “Yellow versus Green”. The colored cells correspond to the color-specific DEGs that can be found in two comparisons, Table S9: Pigment validation by Raman spectroscopy, full results table.
