# Supplementary material for: Molecular Pathways and Pigments Underlying the Colors of the Pearl Oyster Pinctada margaritifera var. cumingii (Linnaeus 1758)
Source: Genes (Basel). 2021 Mar 15;12(3):421. doi: 10.3390/genes12030421 (PMC7998362; doi:10.3390/genes12030421)
Supplement: Supplementary file 1 [file genes-12-00421-s001.zip › Supplementary Materials Protocol S1 and S2.docx]

**Supplementary Materials Protocol**

**Protocol S1:** Detailed parameters of the bioinformatic pipelines.

**Protocol S2:** Detailed protocol of the homology modeling.

**Protocol S1:** Detailed parameters of the bioinformatics pipelines

- The FastQC program (V0.11.5) ([www.bioinformatics.babraham.ac.uk/projects/fastqc](http://www.bioinformatics.babraham.ac.uk/projects/fastqc)) was used to check the quality of raw reads. In order to check the .fastqc files more easily and quickly, we used the *ReadFastQC* R package (https://github.com/PLStenger/ReadFastQC).

- Parameters for Trimmomatic (V0.36.4): PE -phred33 input_forward.fq.gz input_reverse.fq.gz output_forward_paired.fq.gz output_forward_unpaired.fq.gz output_reverse_paired.fq.gz output_reverse_unpaired.fq.gz ILLUMINACLIP:TruSeq3-PE.fa:2:30:10 LEADING:3 TRAILING:26 SLIDINGWINDOW:4:26 MINLEN:25

- Parameters for TopHat (V1.4.0): --library-type FR First Strand -r 45 --mate-std-dev 20 -a 8 -I 20,000. We set maximum intron length at 20,000 bp because, according to Takeushi et al. (2012), the average intron length for *P. fucata* is 4815 bp; so TopHat was tested with 500 000 bp (default), 100,000 bp and 10,000 bp but the amount of proper pairing did not change significantly (84.29%, 84.29%, and 84.10%).

- In order to assess the success of the mapping, FlagStat (V2.0) was run and information was extracted with the *ReadFlagStat* R package (<https://github.com/PLStenger/ReadFlagStat>) to better visualize information between individuals.

- Parameters for Cufflinks (V2.2.1.0): -m 45, -s20, -I 20,000, -F 0.1, -j 8, -G

-Parameters for Cuffmerge (V2.2.1.0): The files were simple merged together from several Cufflinks assemblies.

- Parameters for HTSeq-count (V0.6.1): -m union –s yes –a 10

- Parameters for RBGOA: In order to obtain more precise GO with a high number of genes the parameters used for the clustering were: largest 0.1, smallest 1, clusterCutHeight 0.1, absValue = -log(0.05,10). The custom RBGOA plots were performed with the custom gomwuPlot function (<https://github.com/PLStenger/RBGOA_custom_graphics>).

- Scripts for PLASTx or PLASTp sequence comparison and InterPro-scan**:**

The following sripts contains scripts material to run (Author: P. Durand, Oct 2018):

- PLASTx or PLASTp sequence comparison

- InterPro-scan

jobs in the context of a proteome or genome annotation.

###########################################################################

###########################################################################

In directory named: 01-prepare-fasta

###########################################################################

###########################################################################

###########################################################################

File named: readme.txt

###########################################################################

Use of this script:

This script can be used to slice a large FASTA files into smaller ones.

To be used to run parallel PLAST and IPRscan jobs on each FASTA slice.

1. edit script cut-fasta.pbs (REQUIRED):

lines 7-17

2. run:

qsub cut-fasta.pbs

###########################################################################

File named: cut-fasta.pbs

###########################################################################

#!/usr/bin/env bash

#PBS -q sequentiel

#PBS -l mem=2g

#PBS -l walltime=01:00:00

# ##################################################################

# BEGIN: Data to process

# edit this part as needed to handle YOUR Fasta files!

# FASTA file to cut into smaller slices

WORKDIR="$DATAWORK/tuto-bioinfo/pmarg-annotation/data"

DATAFILE="transcriptome.fna"

# slice size ; unit: nb sequences

SLICESIZE=500

# END: Data to process

# ###################################################################

# do some file naming stuffs

filename=$(basename -- "$DATAFILE")

extension="${filename##*.}"

filename="${filename%.*}"

# Prepare a TIME specific format string

export TIME="U=%U;S=%S;E=%E;P=%P;M=%M;K=%K;F=%F;W=%W;I=%I;O=%O"

TIMEFNAME=${WORKDIR}/cut-${filename}.time

# Load JAVA environment requested by PLAST

. /etc/profile.d/modules.sh

module load java/1.8.0_121

# Set cut command to use

CUTTOOL="/appli/bioinfo/beedeem-tools/2.0.0/cut.sh"

# Prepare cut command

CMD="/usr/bin/time -o $TIMEFNAME --format='$TIME' $CUTTOOL -w $WORKDIR -i $WORKDIR/$DATAFILE -p $SLICESIZE"

# dump cmd to be executed in PBS log for this job

echo $CMD

# run command

eval $CMD

###########################################################################

###########################################################################

In directory named: 02-plast

###########################################################################

###########################################################################

###########################################################################

File named: readme.txt

###########################################################################

Use of these scripts:

1. edit script 01-xxx.sh (REQUIRED):

lines 21-41

2. run script 01-xxx.sh

3. run script 02-xxx.sh

###########################################################################

File named: 01-generate-plast-scripts.sh

###########################################################################

#!/usr/bin/env bash

# ###

#

# A script aims at generating PLAST execution

# scripts to be used on a scheduler-based computational

# infrastructure.

#

# Use:

#

# ./generate_scripts.sh

#

# Before running this script, please edit values at the

# beginning of this script and, if needed, have a look at:

# ./plast_template.pbs

#

# @author Patrick G. Durand, Ifremer, July 2018

#

# ##################################################################

# BEGIN: Data to process

# edit this part as needed to handle YOUR Fasta files!

# path to fasta files (must exist)

SEQ_FILE_PATH="${DATAWORK}/tuto-bioinfo/pmarg-annotation/data"

# path to PLAST results file (will be created)

RES_FILE_PATH="${DATAWORK}/tuto-bioinfo/pmarg-annotation/results/plast"

# fasta file prefix and suffix used to gather all files

# to run parallel PLAST jobs

SEF_FILE_PREFIX="transcriptome_"

SEQ_FILE_EXT=".fna"

# use either "N" or "P" to specify the content of your files:

# nucleoties or proteins.

SEQ_FILE_TYPE="N"

# Reference bank: either "P" (swissprot) or "PL" (TrEMBL)

# change bank in plast=template.txt

REF_BANK="P"

# END: Data to process

# ###################################################################

# CAUTION: do not edit script below, unless you really know what

# you do!

# File containing list of generated scripts

# It can be used to submit BLAST job by order

# (as generated by this script)

SCRIPTLISTFILE="script_list.txt"

[ -e $SCRIPTLISTFILE ] && rm $SCRIPTLISTFILE

touch $SCRIPTLISTFILE

# File containg the PLAST template: it is used to generate PBS

# script jobs for DATARMOR

FILE="plast_template.txt"

# PLAST comparisons: (see plast_template.txt)

COMP="$SEQ_FILE_TYPE:$REF_BANK"

# Nb. cores to use; beware: updating this value may require

# to update template PBS script

CORE="28"

# Memory to use (unit: Gb)

MEM=115

# Walltime to use (Unit: hours)

WTIME=24

nbscript=0

QTYPE=${COMP%:*}

STYPE=${COMP#*:}

SEQ_FILES=(${SEQ_FILE_PATH}/${SEF_FILE_PREFIX}*${SEQ_FILE_EXT})

for (( j = 0 ; j < ${#SEQ_FILES [@]} ; j++ )) ; do

SEQ_FILE="${SEQ_FILES [j]}"

F_NAME=$(basename -- "$SEQ_FILE")

F_NAME="${F_NAME%.*}"

FSCRIPT=${F_NAME}-${QTYPE}-${STYPE}-${MEM}gb-${CORE}c.pbs

WK_DIR=${RES_FILE_PATH}/${FSCRIPT}

echo "Generating $FSCRIPT ..."

[ -e $FSCRIPT ] && rm $FSCRIPT

touch $FSCRIPT

chmod +x $FSCRIPT

echo "$FSCRIPT" >> $SCRIPTLISTFILE

nbscript=$((nbscript+1))

# Read the template line by line and replace some

# variables (defined in the template) by their

# values as set in this script

while IFS= read -r line

do

line=${line//@CORE@/$CORE}

line=${line//@MEM@/$MEM}

line=${line//@QTYPE@/$QTYPE}

line=${line//@STYPE@/$STYPE}

line=${line//@WTIME@/$WTIME}

line=${line//@WORKINGDIR@/$WK_DIR}

line=${line//@QUERYFILE@/$SEQ_FILE}

echo "$line" >> $FSCRIPT

done < "$FILE"

done

echo ""

echo "$nbscript scripts generated"

echo "You can now use '02-submit-plast-scripts.sh' to submit jobs to PBS"

###########################################################################

File named: 02-submit-plast-scripts.sh

###########################################################################

#!/usr/bin/env bash

# ###

#

# A script to sumbit automatically PLAST jobs generated by

# 01-generate-plast-scripts.sh.

#

# Use:

#

# ./02-submit-plast-scripts.sh

#

# @author Patrick G. Durand, Ifremer, Nov 2017

#

# File containing list of generated scripts

# (file created by generate_scripts.sh)

SCRIPTLISTFILE="script_list.txt"

# File that will contain PBS Job IDs

JOBFILE="job-list.txt"

[ -e $JOBFILE ] && rm $JOBFILE

touch $JOBFILE

nbscriptok=0

nbscriptko=0

while IFS= read -r SCRIPT

do

echo "Submitting $SCRIPT to PBS ... "

JOBID=`qsub $SCRIPT`

if [ [ "$JOBID" == *"datarmor"* ]] ; then

echo " OK: $JOBID"

echo "$SCRIPT:$JOBID" >> $JOBFILE

nbscriptok=$((nbscriptok+1))

else

echo " KO"

nbscriptko=$((nbscriptko+1))

fi

done < "$SCRIPTLISTFILE"

echo ""

echo "$nbscriptok scripts submitted"

echo "$nbscriptko scripts NOT submitted (check KO lines, above)"

echo "Job file is: $JOBFILE"

###########################################################################

File named: plast_template_v2.0.0.txt

###########################################################################

#!/usr/bin/env bash

#PBS -q mpi

#PBS -l select=1:ncpus=@CORE@:mpiprocs=@CORE@:mem=@MEM@g

#PBS -l walltime=@WTIME@:00:00

# Type of query: P(rotein) or N(ucleotide)

Q_TYPE=@QTYPE@

QUERY=@QUERYFILE@

# the working directory: where to put results, logs, etc.

WK_DIR="@WORKINGDIR@"

mkdir -p $WK_DIR

# envvar used by PLAST logging system: must be unique and thread safe

export KL_WORKING_DIR="$WK_DIR"

# Type of subject: P or PS(Protein Small), PL (Protein Large), N or NS (Nucleotide Small) or NL (Nucleotide Large)

S_TYPE=@STYPE@

case "$S_TYPE" in

"P" | "PS")

SUBJECT=/XXXXXXXX/Uniprot_SwissProtM.pal

SUBJECT_NAME=swiss

;;

"PL")

SUBJECT=/XXXXXXXX/Uniprot_TrEMBLM.pal

SUBJECT_NAME=trembl

;;

*)

echo "ERROR: unknown subject type: $S_TYPE"

exit 1

;;

esac

# PLAST output format

# 1:tabular ; 2: extended tabular ; 4: NCBI XML

FORMAT=4

case "$FORMAT" in

"1" )

FORMAT_EXT="tab"

;;

"2" )

FORMAT_EXT="etab"

;;

"4" )

FORMAT_EXT="xml"

;;

esac

# PLAST command to use

PLAST_HOME=/XXXXXXXX/beedeem-tools/2.0.0

PLAST_CMD="?"

QT=${Q_TYPE:0:1}

ST=${S_TYPE:0:1}

if [ "$QT" == "P" ] && [ "$ST" == "P" ]; then

PLAST_CMD="plast.sh -p plastp"

PLAST_CMD_NAME="plastp"

elif [ "$QT" == "N" ] && [ "$ST" == "P" ]; then

PLAST_CMD="plast.sh -p plastx"

PLAST_CMD_NAME="plastx"

fi

# Output file name

F_NAME=$(basename -- "$QUERY")

F_NAME="${F_NAME%.*}"

OUTFNAME=$WK_DIR/${F_NAME}

PTIMEFNAME=${OUTFNAME}-plast.time

ATIMEFNAME=${OUTFNAME}-annot.time

PLASTFNAME=${OUTFNAME}.${FORMAT_EXT}

ANNOTFNAME=${OUTFNAME}.zml

# Prepare a TIME specific format string

export TIME="U=%U;S=%S;E=%E;P=%P;M=%M;K=%K;F=%F;W=%W;I=%I;O=%O"

# Load JAVA environment requested by PLAST

. /etc/profile.d/modules.sh

module load java/1.8.0_121

# Prepare PLAST command

CMD="/usr/bin/time -o $PTIMEFNAME --format='$TIME' $PLAST_HOME/$PLAST_CMD -i $QUERY -d $SUBJECT -o $PLASTFNAME -maxhits 20 -maxhsps 1 -e 1e-3 -a @CORE@ -seeds 1"

# dump cmd to be executed in PBS log for this job

echo $CMD

# eventually, run PLAST!

eval $CMD

# This can be used to produce Accesion IDs compatible with B2G.

if [ $FORMAT == 4 ]; then

PLASTFNAME_2=${OUTFNAME}-accs-b2g.${FORMAT_EXT}

# convert this line:

# <Hit_accession>gi|1207922646|ref|XP_021350314.1|</Hit_accession>

# to this line:

# <Hit_accession>XP_021350314</Hit_accession>

cat $PLASTFNAME | sed -e "s/\(<Hit_accession>\) [^|]*| [^|]*| [^|]*|\( [^.]*\)\(\..*\)\{0,1\}|\(<\/Hit_accession>\)/\1\2\4/g" > $PLASTFNAME_2

fi

# Annotation: full

#ANNOT_TOOL="/XXXXXXXX/beedeem/latest/annotate.sh"

#CMD="/usr/bin/time -o $ATIMEFNAME --format='$TIME' $ANNOT_TOOL -i $PLASTFNAME -o $ANNOTFNAME -type full -writer zml"

# dump cmd to be executed in PBS log for this job

#echo $CMD

# run annotation task

#eval $CMD

###########################################################################

###########################################################################

In directory named: 03-iprscan

###########################################################################

###########################################################################

###########################################################################

File named: readme.txt

###########################################################################

Use of these scripts:

1. edit script 01-xxx.sh (REQUIRED):

lines 21-41

2. edit script (optional):

line OUTPUT_TYPE=xml,html

line DOMAINS_SCAN="-appl Pfam -app ProSiteProfiles -app SignalP_EUK"

3. run script 01-xxx.sh

4. run scrun 02-xxx.sh

###########################################################################

File named: 01-generate-ipr-scripts.sh

###########################################################################

#!/usr/bin/env bash

# ###

#

# A script aims at preparing IPR scan job submission PBS scripts.

#

# Use:

#

# ./01-generate-ipr-scripts.sh

#

# Before running this script, please edit values at the

# beginning of this script and, if needed, have a look at:

# ./ipr_template.txt

#

# @author Patrick G. Durand, Ifremer, July 2018

#

# ##################################################################

# ##################################################################

# BEGIN: Data to process

# edit this part as needed to handle YOUR Fasta files!

# path to fasta files (must exist)

SEQ_FILE_PATH="${DATAWORK}/tuto-bioinfo/pmarg-annotation/data"

# path to IPRscan results file (will be created)

RES_FILE_PATH="${DATAWORK}/tuto-bioinfo/pmarg-annotation/results/iprscan"

# fasta file prefix and suffix used to gather all files

# to run parallel IPRscan jobs

SEF_FILE_PREFIX="transcriptome_"

SEQ_FILE_EXT=".fna"

# use either "n" or "p" to specify the content of your files:

# nucleoties or proteins.

SEQ_FILE_TYPE="n"

# END: Data to process

# ###################################################################

# CAUTION: do not edit script below, unless you really know what

# you do!

# File containing list of generated scripts

# It can be used to submit BLAST job by order

# (as generated by this script)

SCRIPTLISTFILE="script_list.txt"

[ -e $SCRIPTLISTFILE ] && rm $SCRIPTLISTFILE

touch $SCRIPTLISTFILE

# File containg the IPR scan template: it is used to generate PBS

# script jobs for DATARMOR

FILE="ipr_template.txt"

SEQ_FILES=(${SEQ_FILE_PATH}/${SEF_FILE_PREFIX}*${SEQ_FILE_EXT})

for (( j = 0 ; j < ${#SEQ_FILES [@]} ; j++ )) ; do

SEQ_FILE="${SEQ_FILES [j]}"

F_NAME=$(basename -- "$SEQ_FILE")

F_NAME="${F_NAME%.*}"

FSCRIPT=${F_NAME}-ipscan.pbs

WK_DIR=${RES_FILE_PATH}/${FSCRIPT}

echo "Generating $FSCRIPT ..."

[ -e $FSCRIPT ] && rm $FSCRIPT

touch $FSCRIPT

chmod +x $FSCRIPT

echo "$FSCRIPT" >> $SCRIPTLISTFILE

nbscript=$((nbscript+1))

# Read the template line by line and replace some

# variables (defined in the template) by their

# values as set in this script

while IFS= read -r line

do

line=${line//@WORKINGDIR@/$WK_DIR}

line=${line//@QUERYFILE@/$SEQ_FILE}

line=${line//@QTYPE@/$SEQ_FILE_TYPE}

echo "$line" >> $FSCRIPT

done < "$FILE"

done

echo ""

echo "$nbscript scripts generated"

echo "You can now use '02-submit-ipr-scripts.sh' to submit jobs to PBS"

###########################################################################

File named: 02-submit-ipr-scripts.sh

###########################################################################

#!/usr/bin/env bash

# ###

#

# A script to sumbit automatically IPRScan jobs generated by

# 01-generate-ipr-scripts.sh.

#

# Use:

#

# ./02-submit-ipr-scripts.sh

#

# @author Patrick G. Durand, Ifremer, July 2018

#

# File containing list of generated scripts

# (file created by generate_scripts.sh)

SCRIPTLISTFILE="script_list.txt"

# File that will contain PBS Job IDs

JOBFILE="job-list.txt"

[ -e $JOBFILE ] && rm $JOBFILE

touch $JOBFILE

nbscriptok=0

nbscriptko=0

while IFS= read -r SCRIPT

do

echo "Submitting $SCRIPT to PBS ... "

JOBID=`qsub $SCRIPT`

if [ [ "$JOBID" == *"datarmor"* ]] ; then

echo " OK: $JOBID"

echo "$SCRIPT:$JOBID" >> $JOBFILE

nbscriptok=$((nbscriptok+1))

else

echo " KO"

nbscriptko=$((nbscriptko+1))

fi

done < "$SCRIPTLISTFILE"

echo ""

echo "$nbscriptok scripts submitted"

echo "$nbscriptko scripts NOT submitted (check KO lines, above)"

echo "Job file is: $JOBFILE"

###########################################################################

File named: ipr_template.txt

###########################################################################

#!/usr/bin/env bash

#PBS -q omp

#PBS -l ncpus=8

#PBS -l mem=32g

#PBS -l walltime=24:00:00

# Notice:

# 1- ncpus: increasing 'ncpus' requires to update 'interproscan.properties'

# system file of InterProScan software. You cannot do that as a

# datarmor user, please contact administrator.

# 2- walltime: you may increase value depending on (1) input sequence file

# and (2) how many domains families you have to scan for.

########################################

## User data ##

########################################

# Set here the full path to your sequence file

USER_FASTA_FILE=@QUERYFILE@

# Set the sequence file type; use either n or p for nucleotide or protein type, respectively.

USER_FILE_TYPE=@QTYPE@

# Set output file format for IPR Scan report.

# Accepted values: tsv, xml, json, html or gff3.

# Comma separated list accepted, e.g.: gff3,xml

OUTPUT_TYPE=xml,html

########################################

## Domains to search for ##

########################################

# As stated by IPRScan documentation, searching for domains requires

# to use some applications. Using 'DOMAINS_SCAN' variable below, setup which

# applications you want to use... and have in mind that the more you use,

# the more you have to wait for results!

DOMAINS_SCAN="-appl Pfam -app ProSiteProfiles -app SignalP_EUK"

# Hint: to save time, DO NOT run a full IPR scan. Instead, only

# scan selected applications, as above.

# It is worth noting that PANTHER and Phobius are not available

# on DATARMOR.

# All others are: TIGRFAM, SFLD, SignalP_GRAM_NEGATIVE,

# SUPERFAMILY, Gene3D, Hamap, Coils, ProSiteProfiles,

# SMART, CDD, PRINTS, ProSitePatterns, Pfam, SignalP_EUK,

# ProDom, MobiDBLite, PIRSF, SignalP_GRAM_POSITIVE, TMHMM

########################################

## Manage script history ##

########################################

# do not modify this section, unless you know what you do!

OUT_FOLDER=@WORKINGDIR@/RES

LOG_DIR=@WORKINGDIR@/LOG

mkdir -p $OUT_FOLDER

mkdir -p $LOG_DIR

cp $0 @WORKINGDIR@

########################################

## Load Java and Blast envs ##

########################################

source /usr/share/Modules/3.2.10/init/bash

module purge

module load java/1.8.0

. /appli/bioinfo/blast/2.6.0/env.sh

########################################

## Prepare and run IPScan ##

########################################

cd $OUT_FOLDER

IPR_DIR=/XXXXXXXXX/interproscan-5.28-67.0/

# Uncomment these two lines to get IPRScan help

#$IPR_DIR/interproscan.sh > $LOG_DIR/ipr_help.log

#exit 0

# Prepare a TIME specific format string

export TIME="U=%U;S=%S;E=%E;P=%P;M=%M;K=%K;F=%F;W=%W;I=%I;O=%O"

TIMEFNAME=@WORKINGDIR@/iprscan.time

# Run an IPR scan job against selected applications

/usr/bin/time -o $TIMEFNAME --format='$TIME' $IPR_DIR/interproscan.sh \

-dp \

-i $USER_FASTA_FILE \

-t $USER_FILE_TYPE \

-f $OUTPUT_TYPE \

-cpu $NCPUS \

$DOMAINS_SCAN \

>> $LOG_DIR/ipr.log

**Protocol S2:** Detailed protocol of the homology modeling

The longest ORFs of some of our candidate genes, which had an FDR < 0.05 and matched with the BlastX result top hit for the sought proteins, were searched for on ORF finder (<https://www.ncbi.nlm.nih.gov/orffinder/>). The ORFs were Blasted for checking. Human reference sequences corresponding to our candidate genes were found in NCBI. The PDB files of the human reference sequence corresponding to our candidate genes and the PDB files corresponding to the longest ORF of each candidate gene were obtained from the I-TASSER (Iterative Threading ASSEmbly Refinement) online service of Zhang Lab (https://zhanglab.ccmb.med.umich.edu/I-TASSER/). On I-TASSER, other information like PDB hit, CscoreEC (the confidence score for the Enzyme Commission number prediction, ranging between [0-1]), active site residues, CscoreLBS (the confidence score of the prediction, ranging from 0 to 1) and ligand binding site residues were also taken.

The modeling of the molecules was done on UCSF Chimera software. The key residues shown in the chimera figures were determined by the comparison of the alignment of the human reference sequence corresponding to our candidate genes with the other longest ORF sequences of our candidate genes, as in Russel et al., 1992 or Lewis et al., 1996. The hinge residues were determined on the HingProt server as in Song et al., 2009.

- Lewis, D. F. V., Eddershaw, P. J., Goldfarb, P. S., & Tarbit, M. H. (1996). Molecular modelling of CYP3A4 from an alignment with CYP102: identification of key interactions between putative active site residues and CYP3A-specific chemicals. Xenobiotica, 26(10), 1067-1086.
- Russell, R. B., & Barton, G. J. (1992). Multiple protein sequence alignment from tertiary structure comparison: assignment of global and residue confidence levels. Proteins: Structure, Function, and Bioinformatics, 14(2), 309-323.
- Song, G., Li, Y., Cheng, C., Zhao, Y., Gao, A., Zhang, R., ... & Liu, Z. J. (2009). Structural insight into acute intermittent porphyria. The FASEB Journal, 23(2), 396-404.
