# Supplementary material for: Molecular Pathways and Pigments Underlying the Colors of the Pearl Oyster Pinctada margaritifera var. cumingii (Linnaeus 1758)
Source: Genes (Basel). 2021 Mar 15;12(3):421. doi: 10.3390/genes12030421 (PMC7998362; doi:10.3390/genes12030421)
Supplement: Supplementary file 1 [file genes-12-00421-s001.zip › Supplementary Materials _Figure_01_Supr_file_01.pdf]

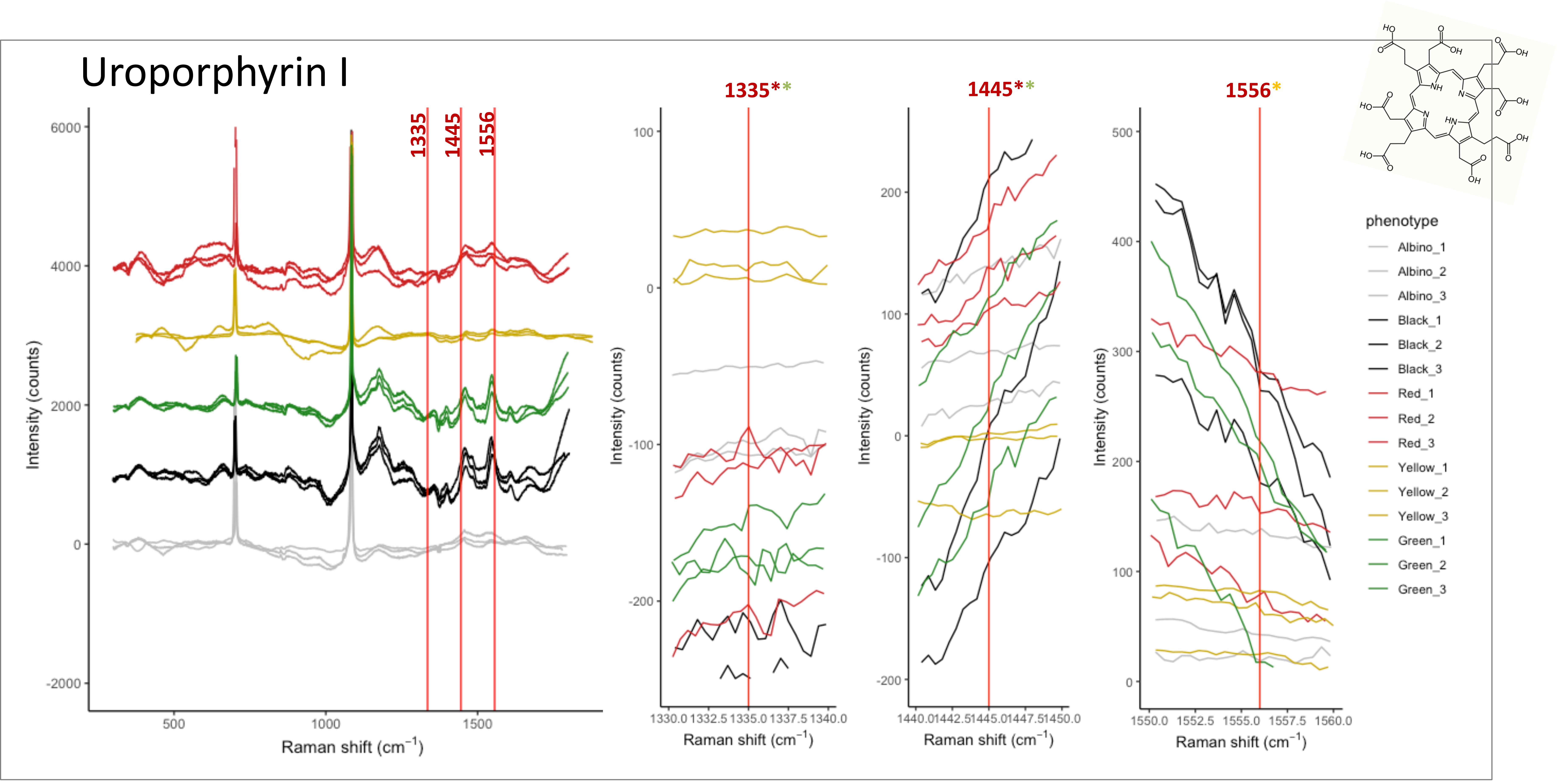
Uroporphyrin I
1335
1445
1556
1335**
1445**
1556*
Intensity (counts)
Raman shift (cm⁻¹)
phenotype
Albino_1
Albino_2
Albino_3
Black_1
Black_2
Black_3
Red_1
Red_2
Red_3
Yellow_1
Yellow_2
Yellow_3
Green_1
Green_2
Green_3

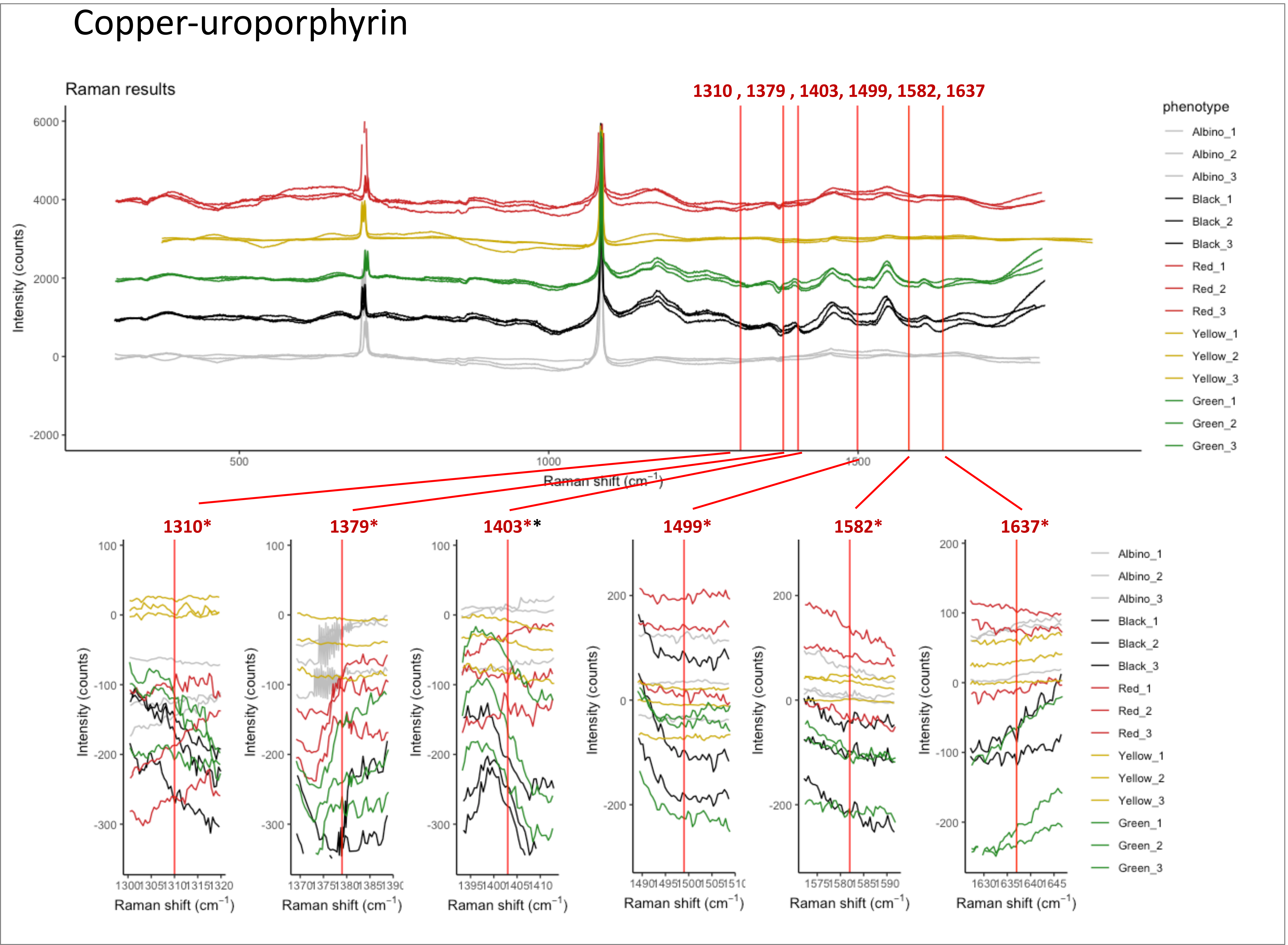
Copper-uroporphyrin
Raman results
1310 , 1379 , 1403, 1499, 1582, 1637
Intensity (counts)
Raman shift (cm⁻¹)
phenotype
Albino_1
Albino_2
Albino_3
Black_1
Black_2
Black_3
Red_1
Red_2
Red_3
Yellow_1
Yellow_2
Yellow_3
Green_1
Green_2
Green_3
1310*
1379*
1403**
1499*
1582*
1637*
