# Supplementary figures and images for: Molecular Pathways and Pigments Underlying the Colors of the Pearl Oyster Pinctada margaritifera var. cumingii (Linnaeus 1758)
Source: Genes (Basel). 2021 Mar 15;12(3):421. doi: 10.3390/genes12030421 (PMC7998362; doi:10.3390/genes12030421)

# Biliverdin

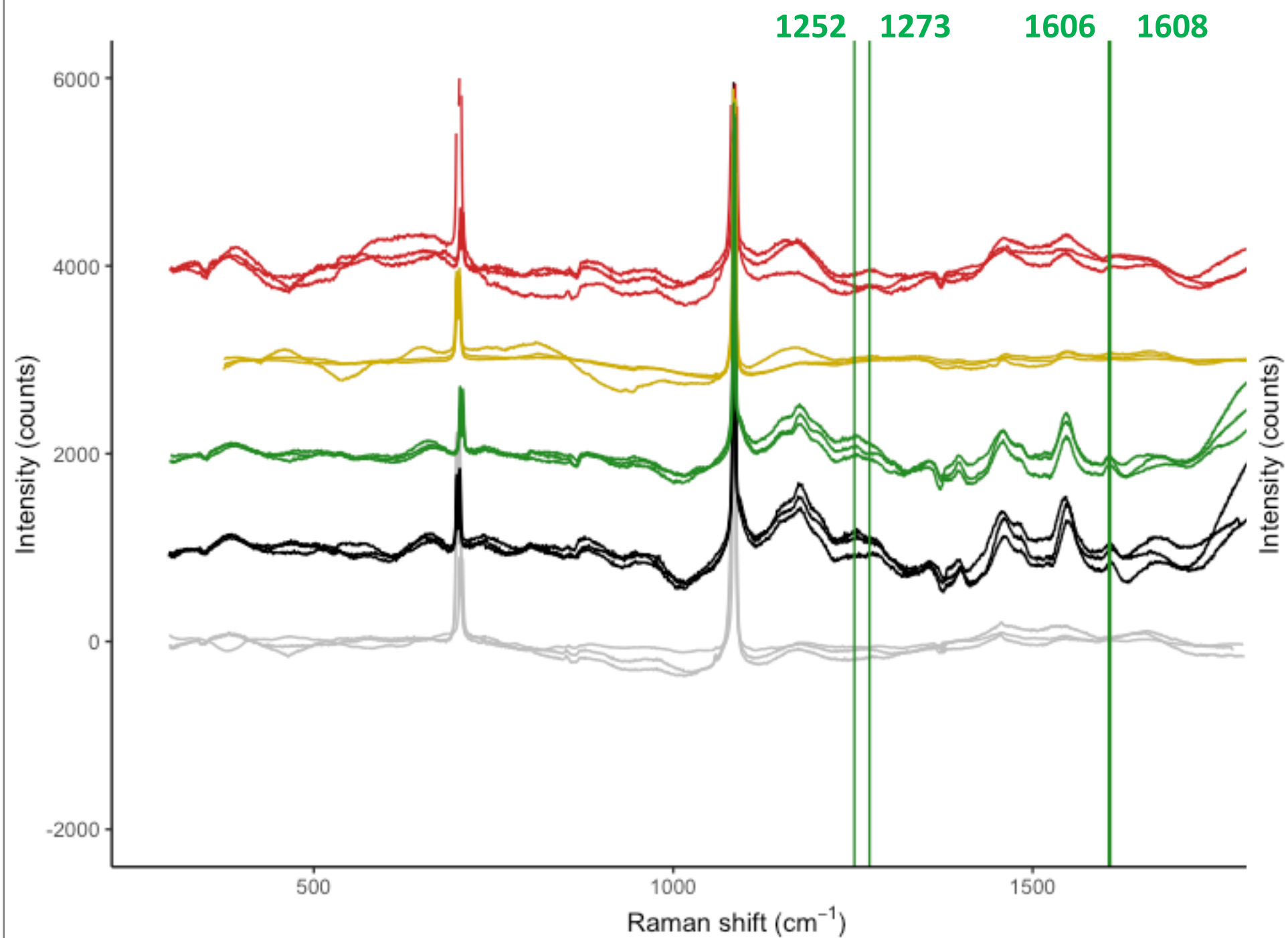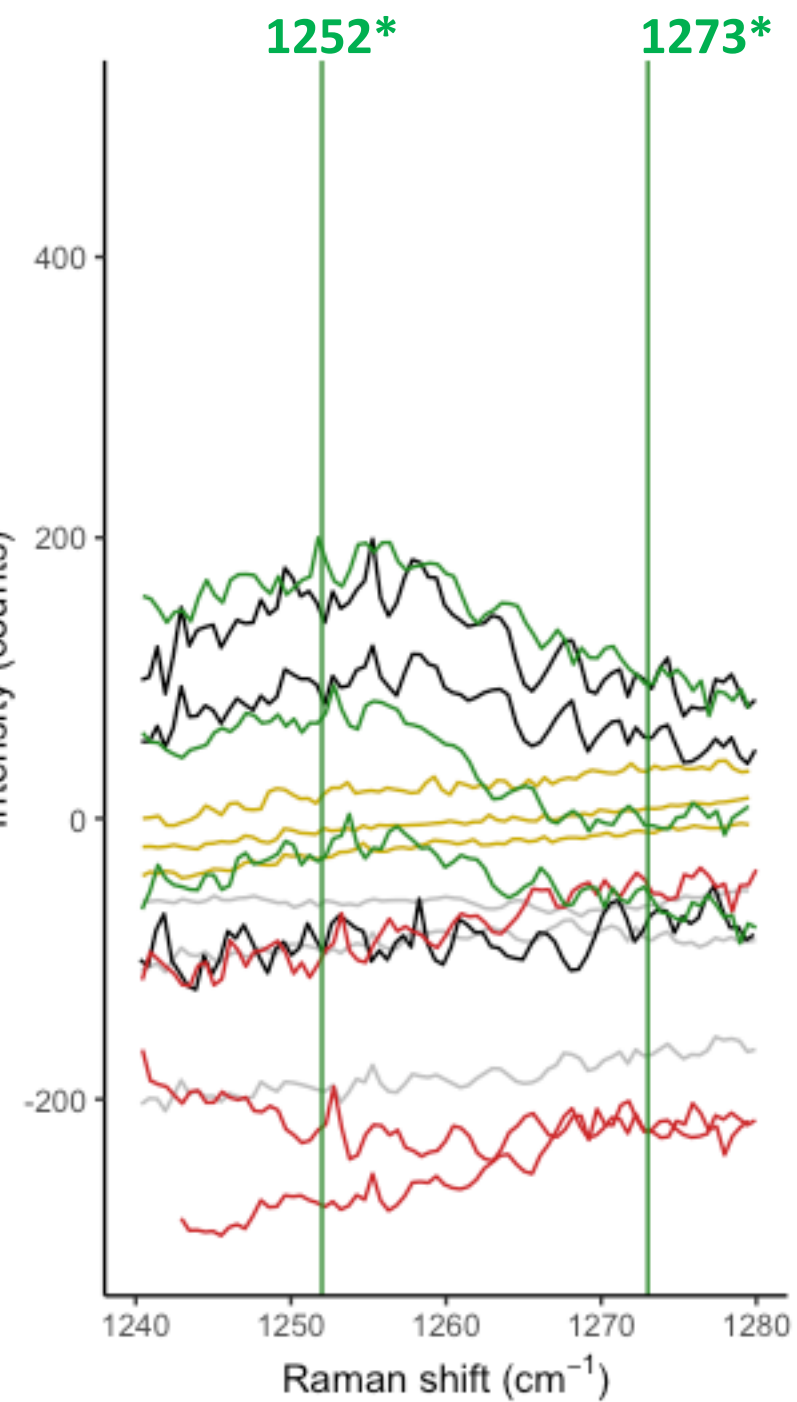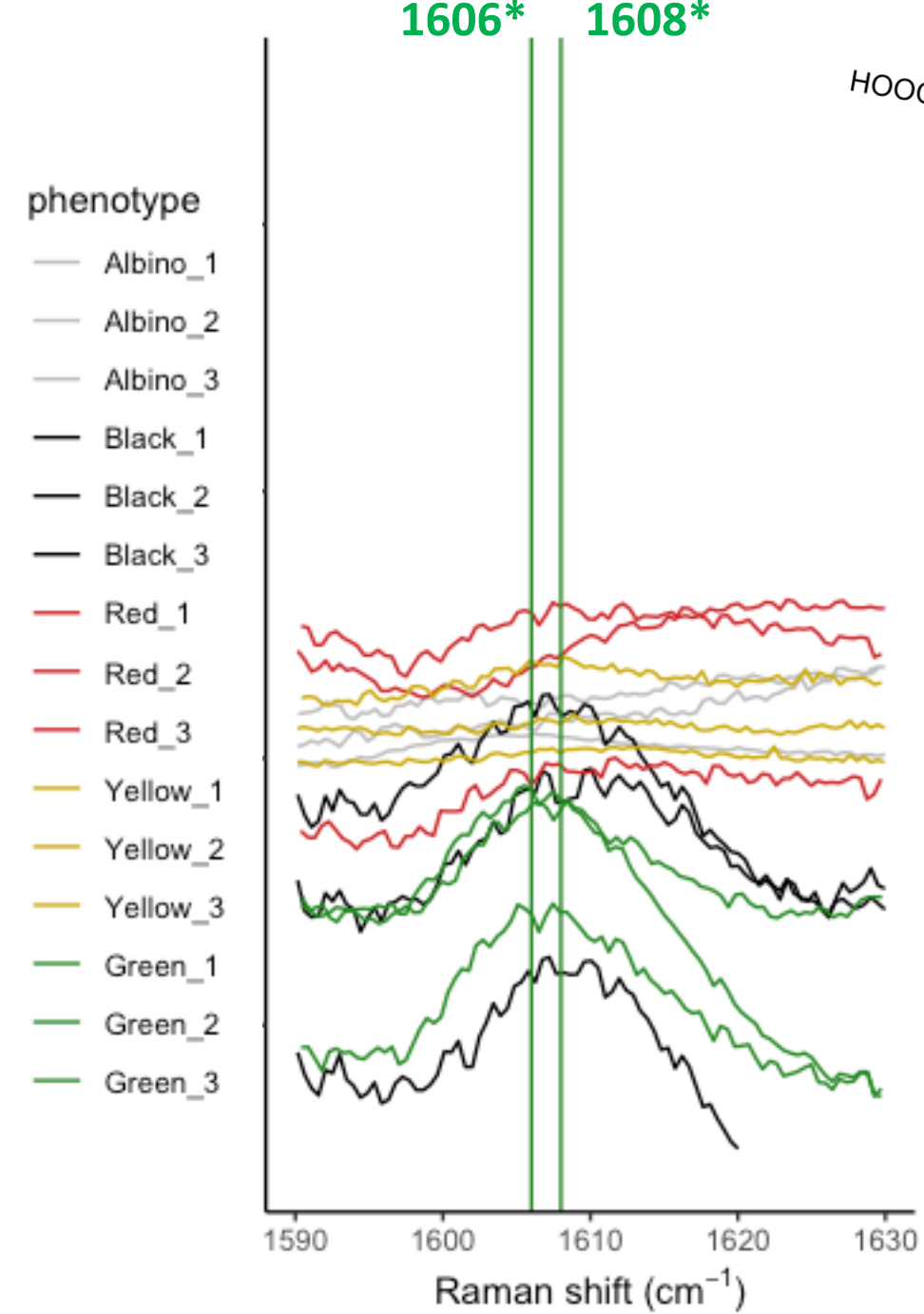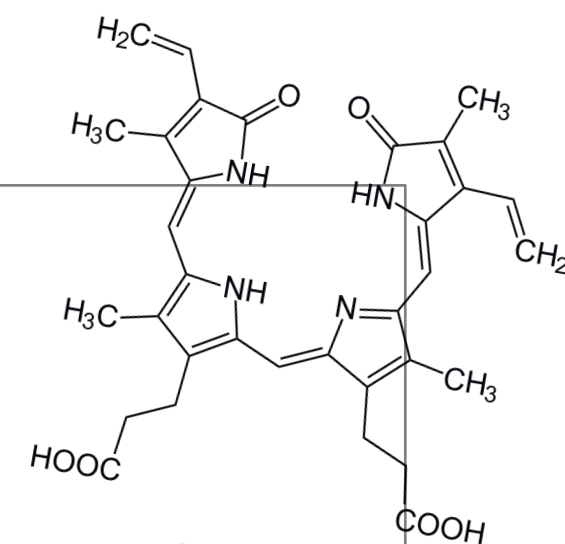

Supplement: Supplementary file 1 [file genes-12-00421-s001.zip › Supplementary Materials _Figure_01_Supr_file_04.pdf]

# Pheomelanin

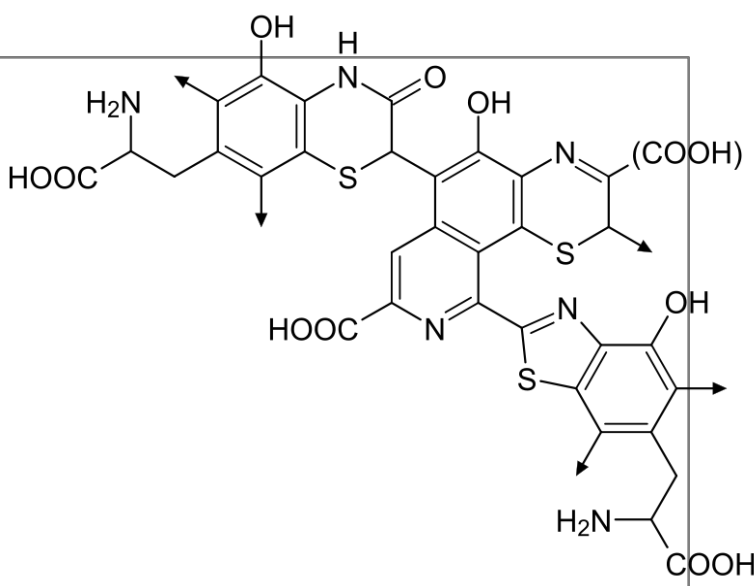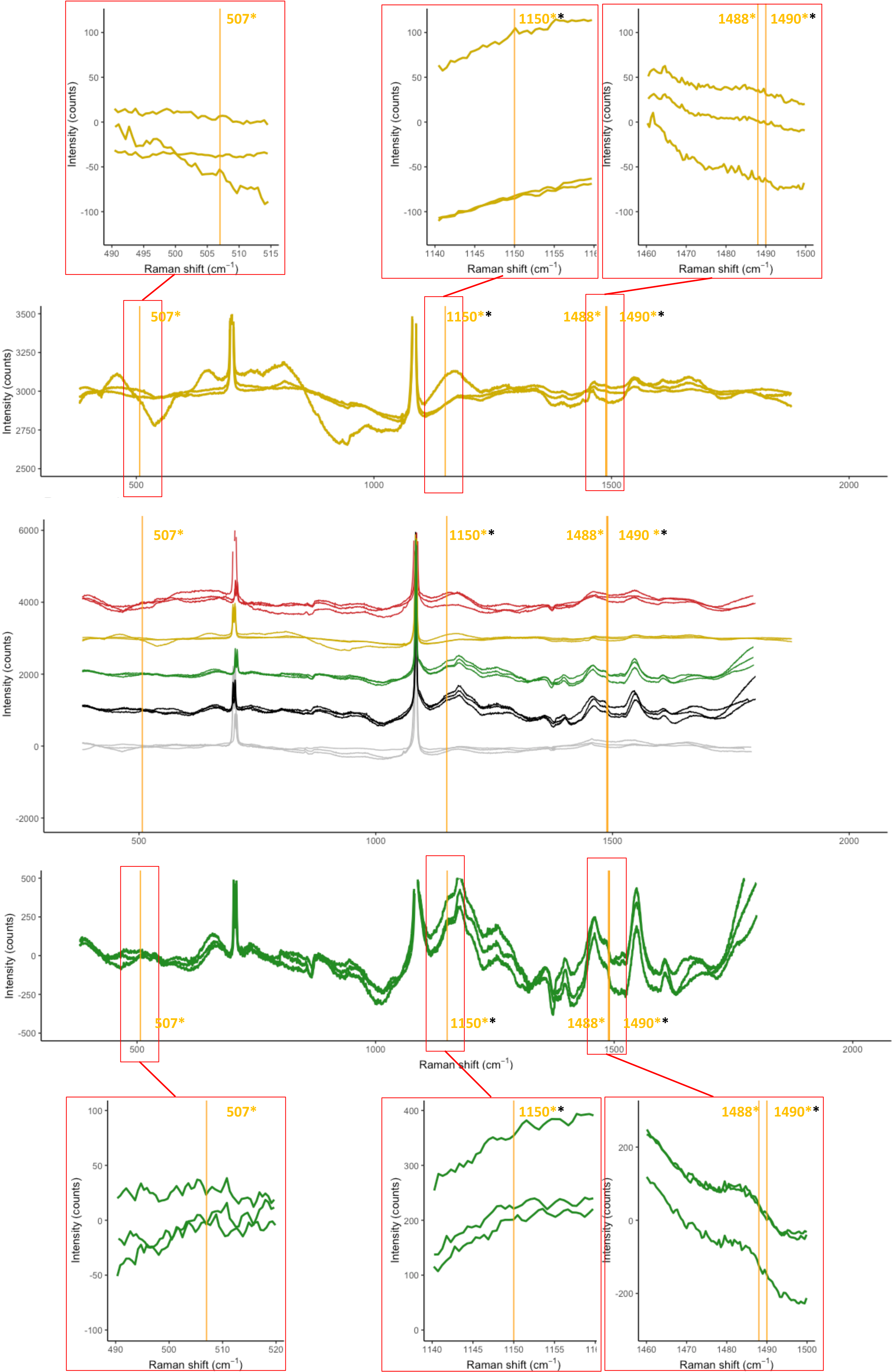

Supplement: Supplementary file 1 [file genes-12-00421-s001.zip › Supplementary Materials _Figure_01_Supr_file_05.pdf]

# Melanin

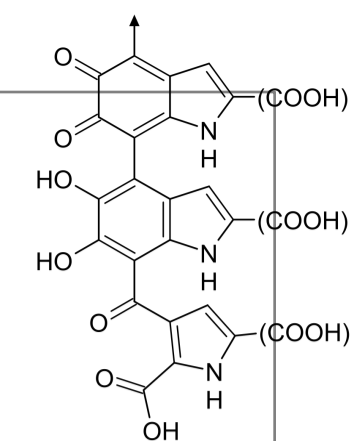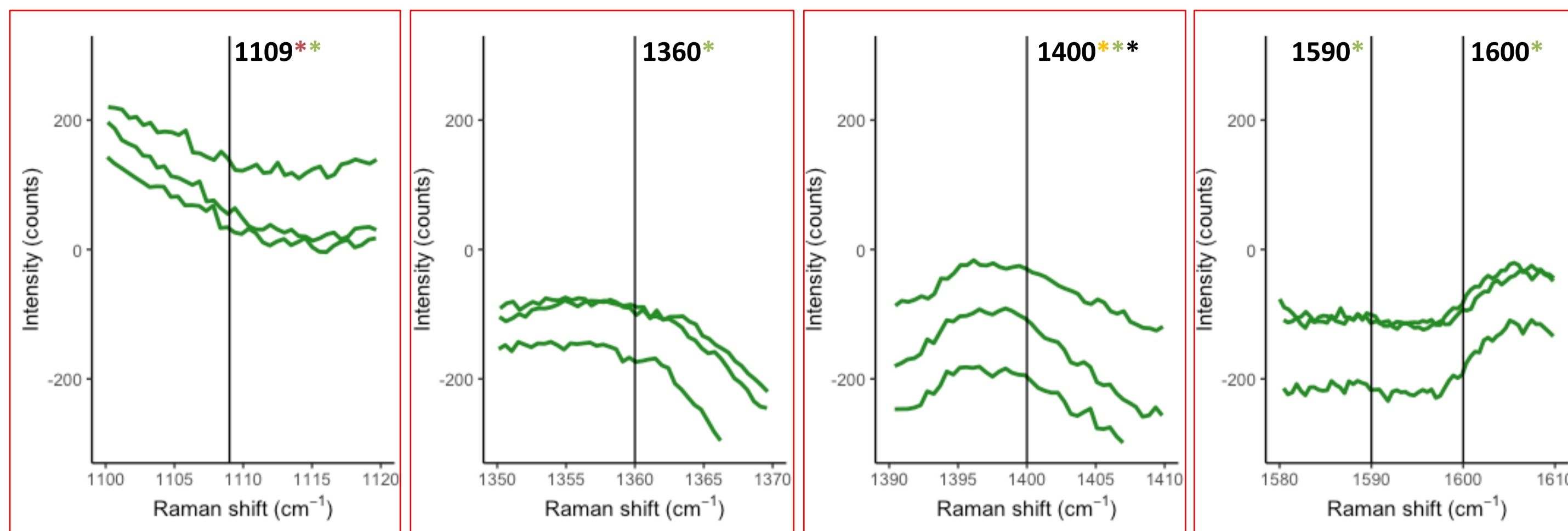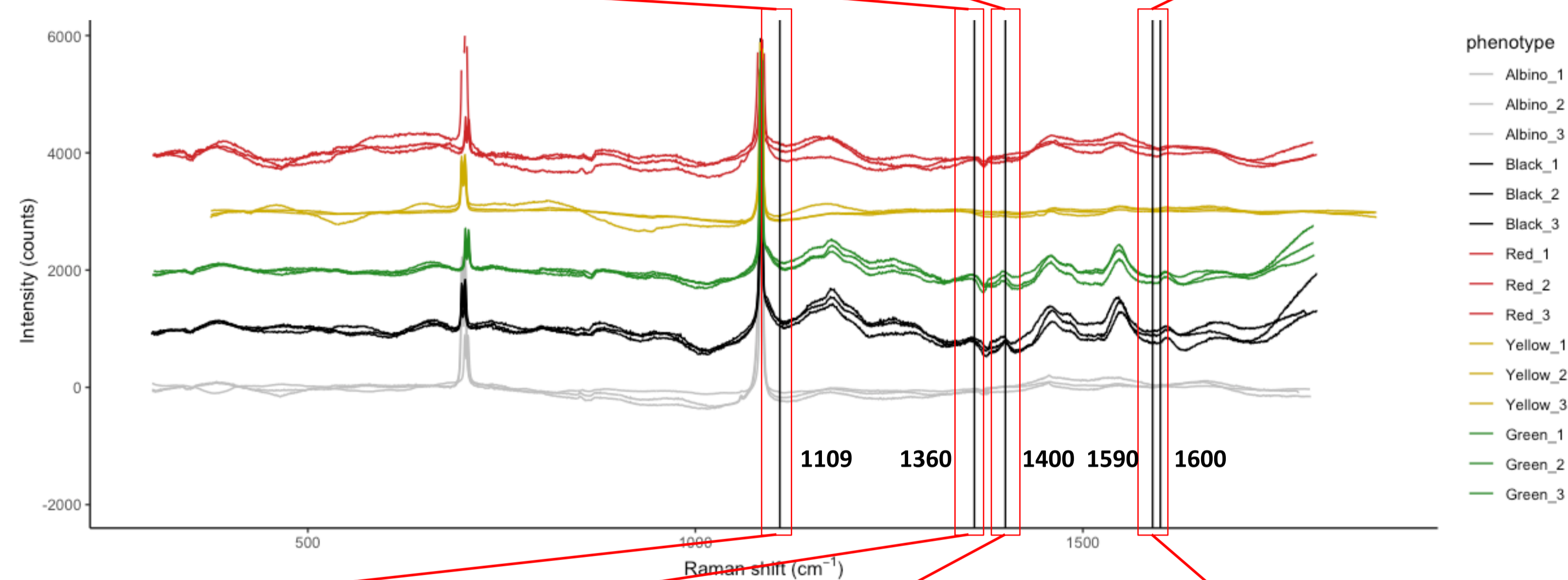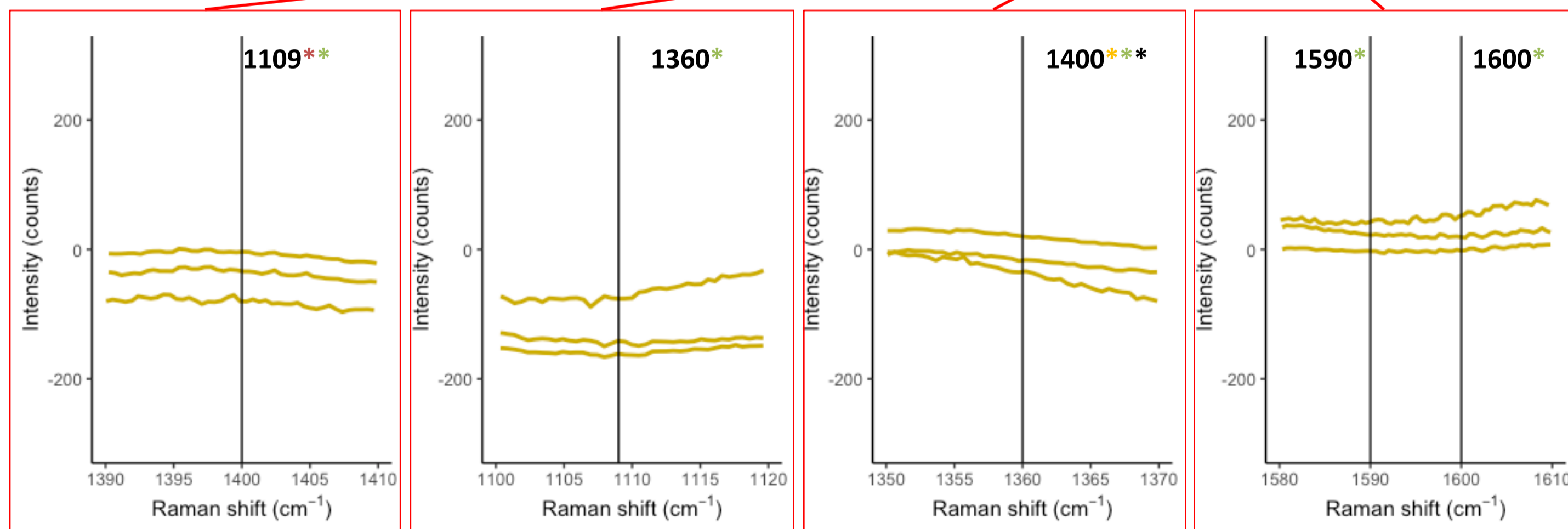

Supplement: Supplementary file 1 [file genes-12-00421-s001.zip › Supplementary Materials _Figure_01_Supr_file_06.pdf]

# Xanthine

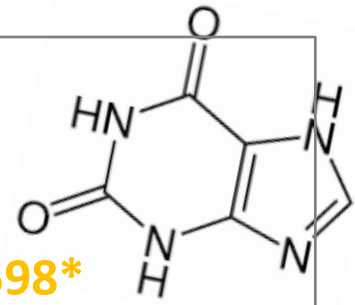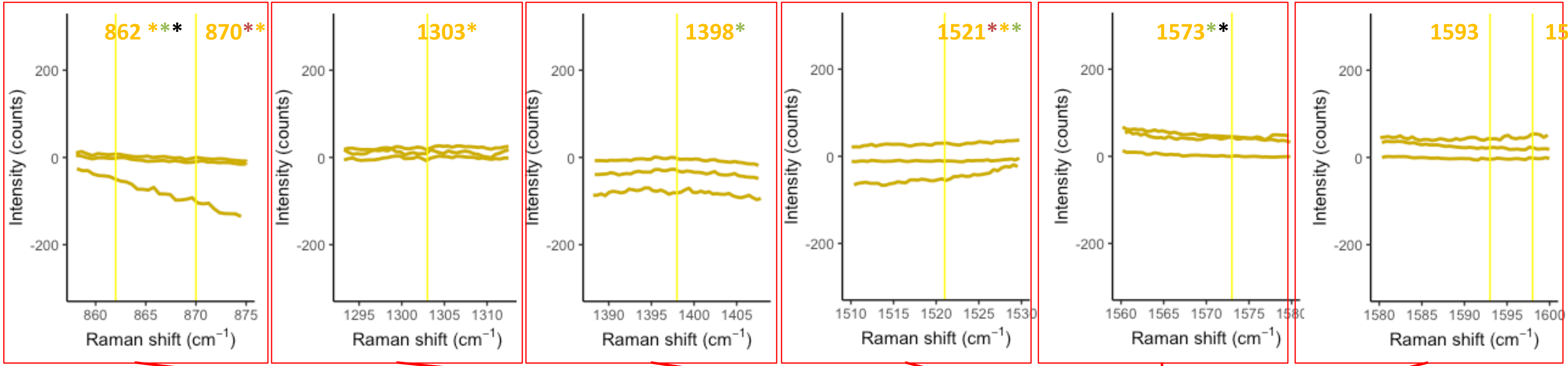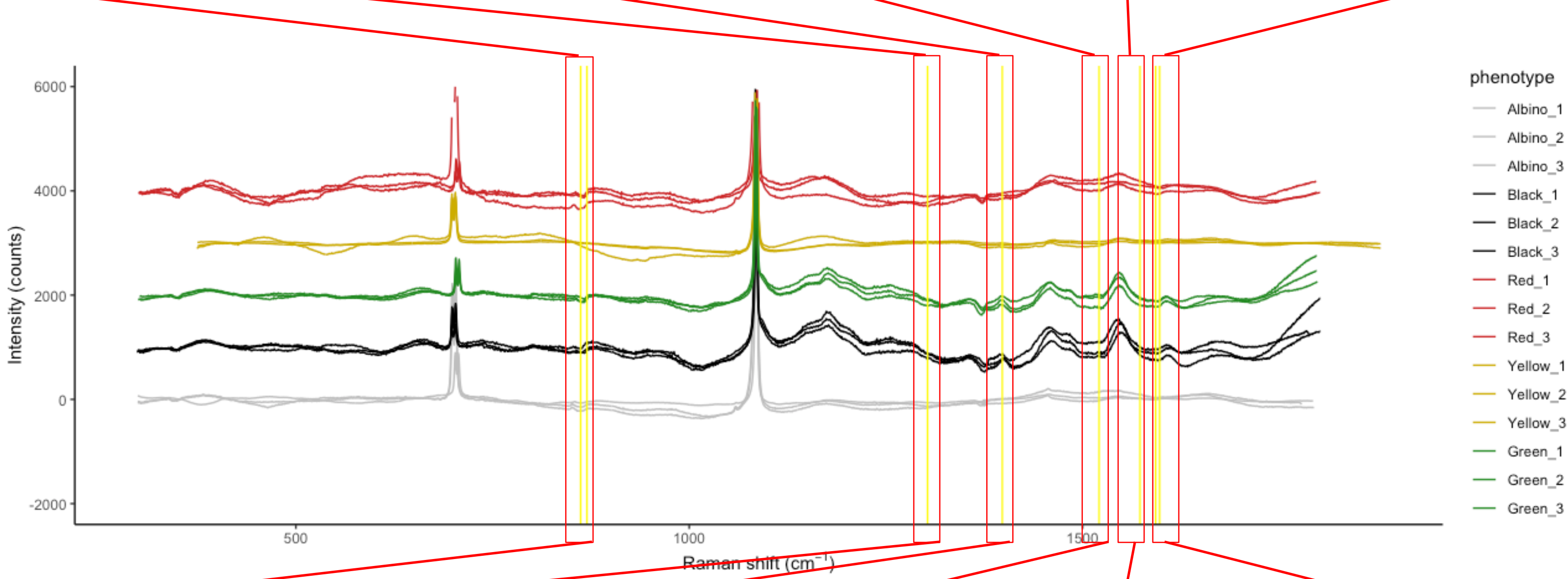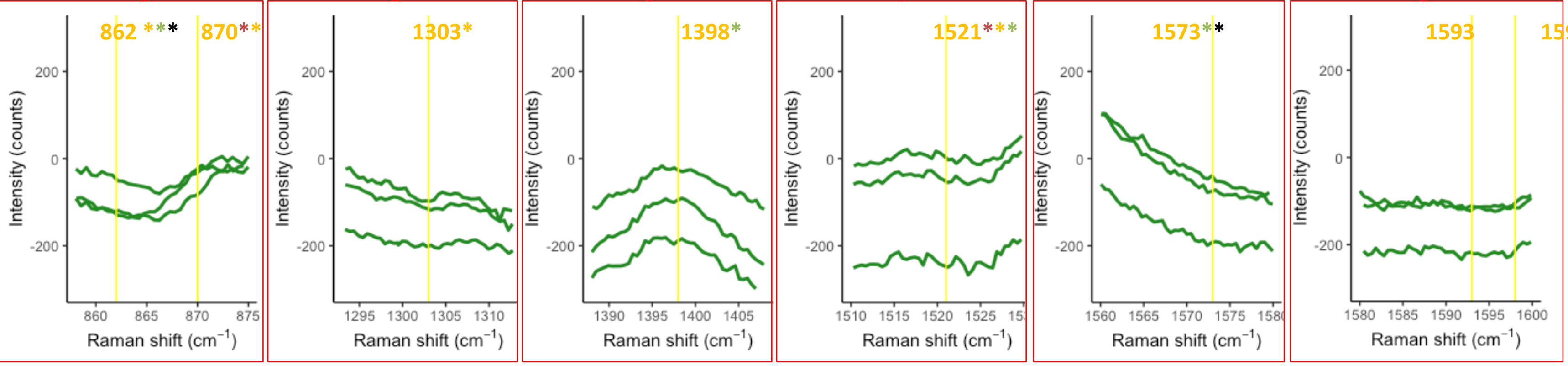

Supplement: Supplementary file 1 [file genes-12-00421-s001.zip › Supplementary Materials _Figure_01_Supr_file_07.pdf]

# Bilirubin

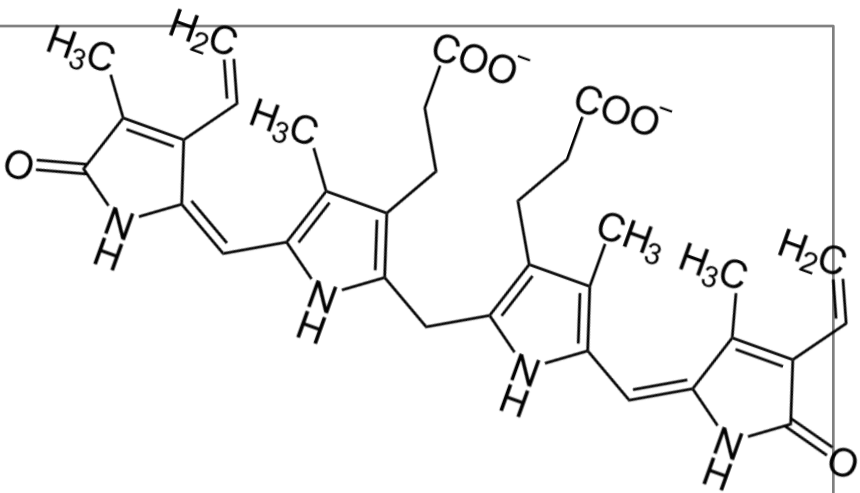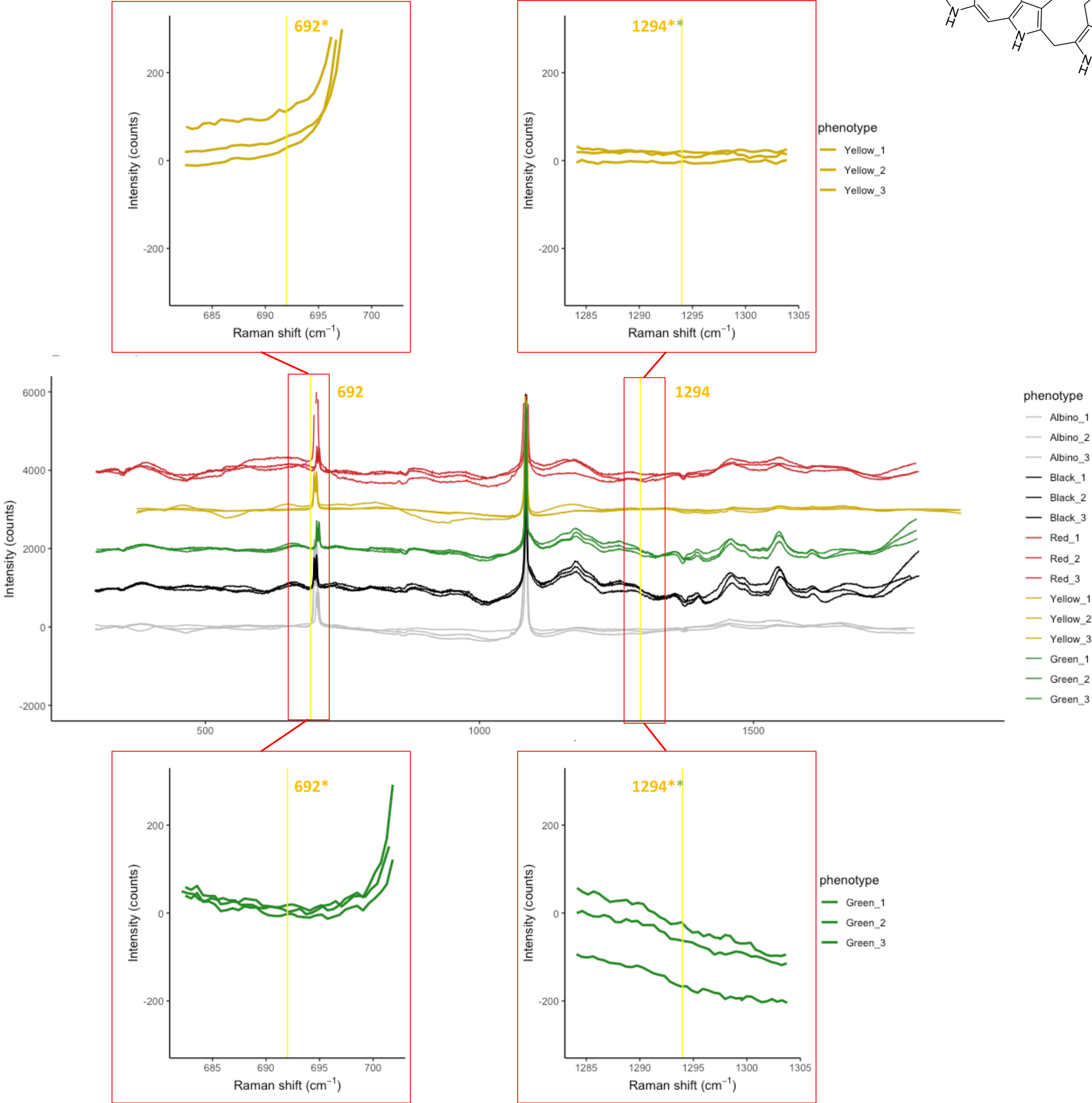

Supplement: Supplementary file 1 [file genes-12-00421-s001.zip › Supplementary Materials _Figure_01_Supr_file_08.pdf]

# FeIII-uroporphyrin

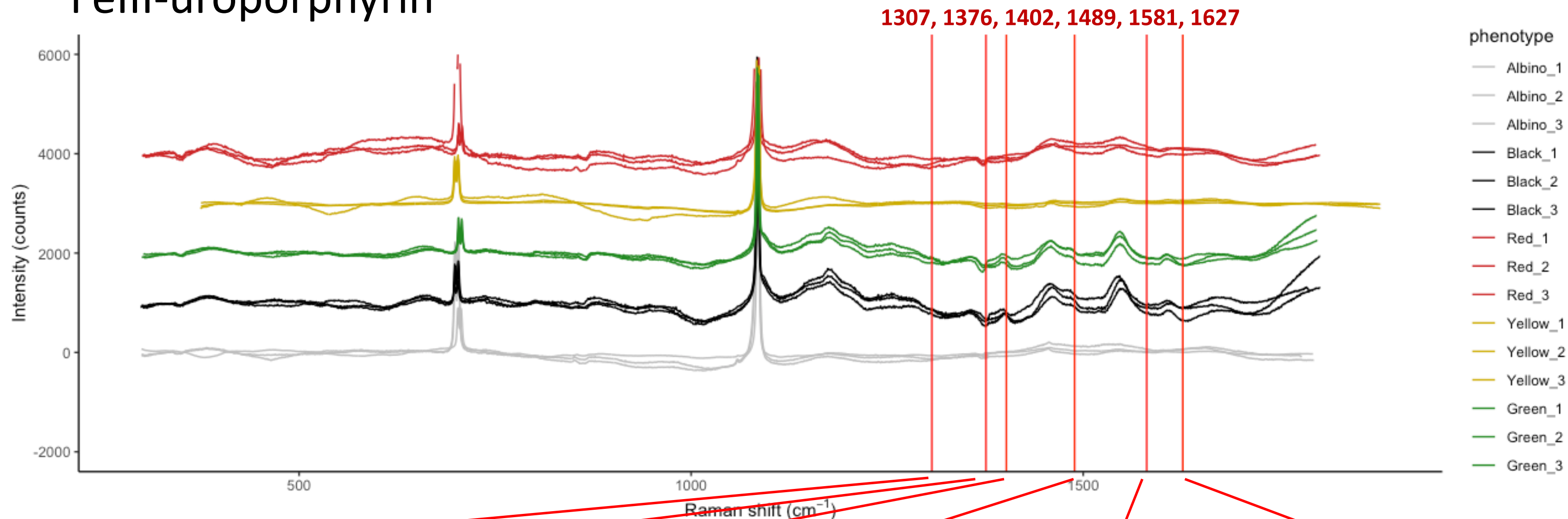

1307\*

1376\*\*

1402\*\*

1489\*\*

1581\*

1627\*

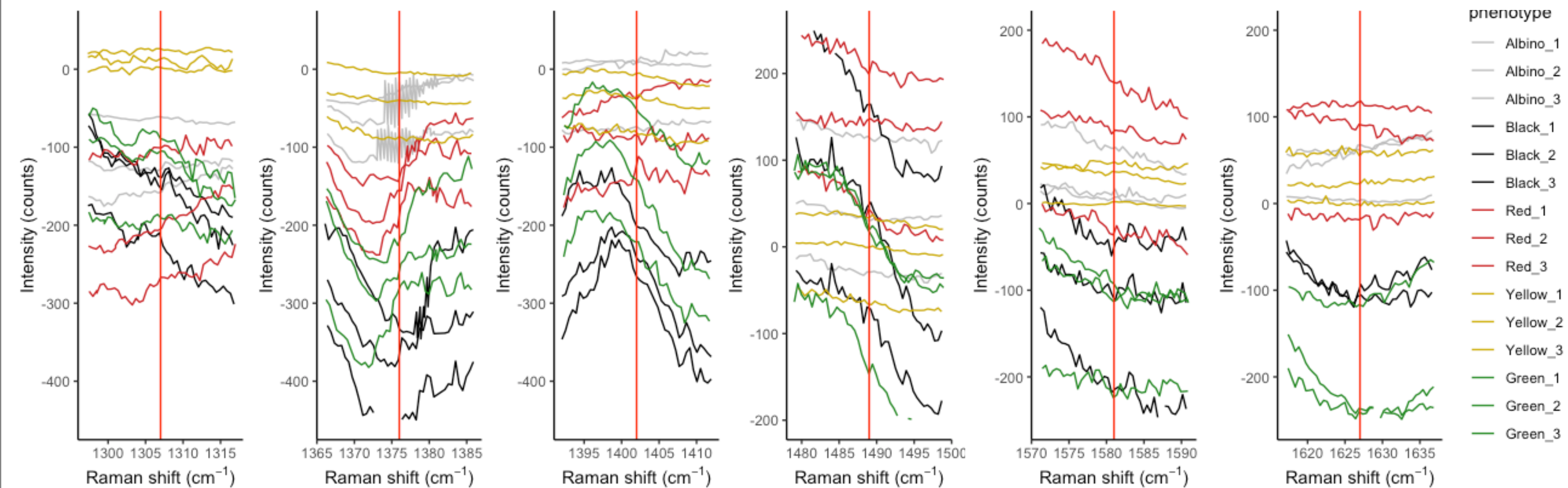

Supplement: Supplementary file 1 [file genes-12-00421-s001.zip › Supplementary Materials _Figure_01_Supr_file_02.pdf]
